# Combined Transcriptome and Metabolome Analyses Reveal Candidate Genes Involved in Tangor (*Citrus reticulata × Citrus sinensis*) Fruit Development and Quality Formation

**DOI:** 10.3390/ijms23105457

**Published:** 2022-05-13

**Authors:** Xiaoyi Bi, Ling Liao, Lijun Deng, Zhenghua Jin, Zehao Huang, Guochao Sun, Bo Xiong, Zhihui Wang

**Affiliations:** 1 College of Horticulture, Sichuan Agricultural University, Chengdu 611130, China; bxy0048@163.com (X.B.); liao19910331@163.com (L.L.); 2020205017@stu.sicau.edu.cn (L.D.); jzh13018205303@163.com (Z.J.); e4203274@163.com (Z.H.); xiongbo1221@sicau.edu.cn (B.X.); 2 Institute of Pomology and Olericulture, Sichuan Agricultural University, Chengdu 611130, China; sunguochao@163.com

**Keywords:** mandarin-orange hybrids, fruit development, quality formation, metabolome, transcriptome

## Abstract

Tangor, an important citrus type, is a hybrid of orange and mandarin and possesses their advantageous characteristics. Fruit quality is an important factor limiting the development of the citrus industry and highly depends on fruit development and ripening programs. However, fruit development and quality formation have not been completely explored in mandarin-orange hybrids. We sequenced the metabolome and transcriptome of three mandarin-orange hybrid cultivars at the early fruiting [90 days after full bloom (DAFB)], color change (180 DAFB), and ripening (270 DAFB) stages. Metabolome sequencing was performed to preliminarily identify the accumulation patterns of primary and secondary metabolites related to fruit quality and hormones regulating fruit development. Transcriptome analysis showed that many genes related to primary metabolism, secondary metabolism, cell wall metabolism, phytohormones, and transcriptional regulation were up-regulated in all three cultivars during fruit development and ripening. Additionally, multiple key genes were identified that may play a role in sucrose, citric acid and flavonoid accumulation, cell wall modification, and abscisic acid signaling, which may provide a valuable resource for future research on enhancement of fruit quality of hybrid citrus. Overall, this study provides new insights into the molecular basis of pulp growth and development regulation and fruit quality formation in mandarin-orange hybrids.

## 1. Introduction

Citrus fruits are popular worldwide for their unique flavor. Among citrus fruits, mandarins (*Citrus reticulata*) are of increasing commercial interest [1]. Mandarin-type fruits are small and easy to peel, and hence, consumer-friendly and responsible for the largest increase in global citrus production [2]. Additionally, mandarins are excellent breeding material, and in recent years, increasing number of citrus hybrids have been bred and are popular with consumers. According to Tanaka’s classification [3], citrus hybrids are divided into two main categories, namely tangor (*Citrus reticulata* × *Citrus sinensis*, i.e., mandarin × orange hybrid) and tangelo (*Citrus paradisi* × *Citrus reticulata*, i.e., grapefruit hybrid × mandarin). Tangor, which possesses the benefits of orange and mandarin, has a flavor similar to mandarin and aroma similar to orange, and has become increasingly popular with consumers in recent years [4,5].

Citrus fruits are highly nutritious, containing primary metabolites, which are an important source of essential nutrients, and secondary metabolites, which exhibit potent health-promoting effects and form an excellent source of bioactive substances [1,6]. Moreover, flavor and texture are the main quality traits of citrus fruits; particularly, they have a unique flavor. Primary and secondary metabolites such as sugars, organic acids, and flavonoids are important components of fruit flavor, while fruit texture is related to cell wall metabolism and lignin synthesis [7,8,9]. In recent years, many studies have been conducted on metabolites during growth and ripening of oranges, mandarins, pomelos, and grapefruits, and the main nutrients and bioactive compounds present in them have been identified [10,11,12,13]. However, as an important hybrid citrus type, little is known about the changes in metabolic levels during fruit development and ripening in tangors. Accumulation of nutrients and phytochemicals in fruits are influenced by different factors (genetic, environmental, agronomic, and cultural practices) and highly dependent on the fruit ripening program [9].

The growth, development, and ripening of citrus fruits is divided into three stages—first involves fruit cell division, that is when the fruit grows slowly; the second is a critical period for growth and forms the cell enlargement period and rapid morphological and physiological changes occur in the absence of cell division; the third stage involves the ripening process, that is when fruit growth declines and the transition to ripening begins, resulting in various changes such as fruit color changes, sugar accumulation, and acid degradation [14]. Fruit growth and ripening are genetically programmed processes that involve physiological, biochemical, molecular, and organoleptic changes [15]. Citrus fruits are characterized by non-leapfrog fruit ripening, and hormones play an important role in the regulation of their fruit growth and development. Currently, techniques such as metabolomics and transcriptomics are being widely used in fruit and plant growth and development studies to reveal different developmental, ripening, and senescence mechanisms [16,17,18]. These techniques have also been widely used to study citrus fruits; for example, Feng et al. performed transcriptome analysis on four tissues of sweet orange fruits at different growth and developmental stages and revealed the expression patterns and metabolic networks of hormones, sucrose, and citric acid during the development of sweet orange [19]. Ding et al. selected fruits of four major citrus fruit varieties at different postharvest stages for metabolomic and transcriptomic analyses and revealed important physiological changes during postharvest storage and characterized the different storage behaviors of different citrus species [20]. In addition, transcriptomic analysis has been performed on three types of grapefruits with different colored flesh to reveal the molecular mechanisms of carotenoid accumulation during their growth and development [21]. However, these studies did not analyze metabolism and transcription during the development and maturation of tangor, one of the mandarin hybrids, and the dynamics of tangor fruit quality formation. Therefore, regulation of growth and development in tangor needs to be studied in detail.

In recent years, the market demand for tangor has been increasing and gradually become a new driving force in the citrus industry. Currently, there are many tangor varieties in the market, but their fruit quality is varies. Therefore, developing, selecting, and breeding relatively higher quality tangor type citrus is still necessary. However, there are few studies on the formation of tangor fruit quality. In this study, pulp tissue of three tangor cultivars (‘Huangguogan’, ‘Orah’ and ‘Daya’) was used as study material. Fruits were sampled during three stages from 90 d to 270 d after full bloom (DAFB)—the fruit expansion stage, 90 DAFB; the color change stage, 180 DAFB, and the ripening stage, 270 DAFB)—which involved the fruit development and ripening stages. Metabolomic and transcriptomic sequencing was performed to obtain metabolite and transcriptome profiles of fruits of the three cultivars during different stages of development. The aim of this study was to analyze the dynamics of metabolites, such as sugars, organic acids, flavonoids, lignans, and hormones, and the major differentially expressed genes (DEGs) associated with fruit quality traits and developmental regulation at different developmental stages of three hybrid citrus cultivars. The results of this study would contribute to our understanding of the molecular basis of quality determination and developmental regulation in tangor fruits. Moreover, it would provide basic data for future research on fruit quality improvement of tangor and help in dealing with the increasing market value.

## 2. Materials and Methods

### 2.1. Sample Collection and Experimental Design

Three tangor cultivars (‘Huangguogan’, ‘Orah’, and ‘Daya’, hereinafter abbreviated as HG, OR, and DY, respectively) were collected from an experimental orchard in Shimian County, Sichuan Province, China (29.23° N, 102.36° E; 780 m above sea level). Three trees per cultivar were selected as one biological replicate. Three biological replicates were used for RNA-seq, and six biological replicates were used for UPLC-MS/MS. The pulp of the fruit at different stages of growth—fruit expansion stage (stage 1, 90 DAFB), the color-conversion stage (stage 2, 180 DAFB), and ripening stage (stage 3, 270 DAFB)—was sampled in liquid nitrogen and then stored at −80 °C for further analysis.

### 2.2. Determination of Fruit Firmness and Sugar and Organic Acid Content

Fruit firmness was measured using TMS-Pilot Precision texture analyzer (FTC, Sterling, VA, USA). The initial force, range of force, puncture speed, and puncture distance were set to 0.75 N, 450 N, 60 mm min^−1^, and 15 mm, respectively.

Sugars were extracted and determined according to the method described by Dong et al. [22]. Sugar content was analyzed using HPLC equipped with a refractive index detector (LC-1260; Agilent Technologies, Sacramento, CA, USA). Samples were isolated on the Innoval NH2 column (4.6 mm × 250 mm, 5 µm, CNW Technologies, Shanghai, China) at room temperature. HPLC was performed using mobile phase (acetonitrile:water = 80:20 (V/V)) isocratic elution with the following parameters: sample volume, 10 μL; flow rate, 1 mL min^−1^ column temperature, 30 °C; detection temperature, 40 °C.

The organic acids were extracted and determined according to the method described by Liao et al. [23]. Organic acids were analyzed using HPLC equipped with a UV detector (LC-1260; Agilent Technologies, Sacramento, CA, USA). Samples were isolated on the C18-WP column (4.6 mm × 250 mm, 5 µm, CNW Technologies, Shanghai, China) at room temperature. HPLC was performed using isocratic elution with mobile phase (4% methanol solution; metaphosphoric acid was used to adjust the pH to 2.6). The parameters were as follows: sample volume, 10 μL; flow rate, 0.8 mL min^−1^; column temperature, 25 °C; detection temperature, 25 °C. Organic acid content was determined by measuring the absorbance at 210 nm. For the determination of sugars and organic acids, three biological replicates were taken and technical replications were performed thrice.

### 2.3. Metabolite Profiling Using UPLC-MS/MS and Data Analysis

Pulp tissue (six biological replicates; 100 mg per replicate) was ground in liquid nitrogen and the homogenate was resuspended in prechilled 500 μL 80% methanol using a vortex. Thereafter, the samples were incubated on ice for 5 min followed by centrifugation at 15,000× *g* and 4 °C for 20 min. LC-MS grade water (255 µL) was added to dilute 500 µL of the supernatant to a final concentration containing 53% methanol. The samples were subsequently transferred to a fresh eppendorf tube and centrifuged at 15,000× *g* and 4 °C for 20 min. Finally, the supernatant was subjected to LC-MS/MS analysis. UHPLC-MS/MS analyses were performed using a Vanquish UHPLC system (Thermo Fisher Scientific, Germany) coupled with an Orbitrap Qexactive TMHF-X mass spectrometer (Thermo Fisher Scientific, Germany) by Novogene Co., Ltd. (Beijing, China). Samples were injected onto a Hypesil Gold column (100 × 2.1 mm, 1.9 μm) using a 17-min linear gradient at a flow rate of 0.2 mL min^−1^. The eluents for the positive polarity mode were eluent A (0.1% Formic acid in water) and eluent B (methanol). The eluents for the negative polarity mode were eluent A (5 mM ammonium acetate, pH 9.0) and eluent B (methanol). The solvent gradient was set as follows: 2% B, 1.5 min; 2–100% B, 12.0 min; 100% B, 14.0 min; 100–2% B, 14.1 min; 2% B, 17 min. QExactiveTMHF-X mass spectrometer was operated in positive/negative polarity mode with spray voltage of 3.2 kV, capillary temperature of 320 °C, sheath gas flow rate of 40 arb, and aux gas flow rate of 10 arb.

The raw data files generated by UHPLC-MS/MS were imported into CD 3.1 library search software for processing, and parameters such as retention time and mass-to-charge ratio were screened for each metabolite. Then set a retention time deviation of 0.2 min and a mass deviation of 5 ppm to align the peaks for different samples to make identification more accurate. Then set the mass deviation 5 ppm, signal intensity deviation 30%, signal-to-noise ratio 3, minimum signal intensity, summation ion and other information for peak extraction, and quantify the peak area at the same time, and then integrate the target ion. The molecular formula was then predicted from molecular ion peaks and fragment ions, and compared with the high-resolution secondary spectra databases mzCloud (https://www.mzcloud.org/, accessed on 3 May 2021), mzVault and Masslist primary databases for metabolite identification. The molecular weight of the metabolite is determined based on the mass-to-charge ratio (*m*/*z*) of the parent ion in the primary mass spectra, and the molecular formula is predicted by the mass number deviation (ppm) and information such as the addition ion, and then matched with the database; the database containing the secondary spectra is matched with the fragment ion and collision energy of each metabolite in the database based on the actual secondary spectra to achieve the secondary identification of the metabolite. QC sample was set up to evaluate the stability of the system during the experiment, and blank sample was used to remove the background ions. The metabolites with Coefficient of Variance (CV) less than 30% [24] in QC samples are then retained as the final identification results for subsequent analysis. The data processing part is based on Linux operating system (CentOS version 6.6) and software R and Python. Statistical analyses were performed using the statistical software R (R version R-3.4.3), Python (Python 2.7.6 version) and CentOS (CentOS release 6.6), When data were not normally distributed, normal transformations were attempted using of area normalization method. These metabolites were annotated using the Kyoto Encyclopedia of Genes and Genomes (KEGG) (https://www.genome.jp/kegg/pathway.html, accessed on 17 February 2022), HMDB (https://hmdb.ca/metabolites, accessed on 18 February 2022), and Lipidmaps database (http://www.lipidmaps.org/, accessed on 17 February 2022). The selection of metabolites for differential accumulation between samples depended mainly on variable importance in projection (VIP), fold change (FC), and *p*-value. VIP refers to the variable projection importance of the first principal component in the partial least squares discriminant analysis (PLS-DA) model [25], representing the contribution of metabolites to population classification. FC is the ratio of the mean of the quantitative values of each metabolite in the group. *p*-values were calculated using *t*-test to indicate significance of differences. In this experiment, threshold values were set to VIP > 1.0, FC > 1.5 or FC < 0.667, and *p*-value < 0.05 [25,26]. Volcano plots were used to filter metabolites of interest based on log2(FC) and -log10(*p*-value) of metabolites determined by ggplot2 in R language. The selected differential metabolites were first annotated on the KEGG database, and the corresponding pathway ratios (the ratio of the number of differential metabolites annotated to the KEGG pathway to the total number of differential metabolites) and number of differential metabolites were then used to determine the enrichment pathways.

### 2.4. RNA Library Preparation and Sequencing

Nine pulp samples (each with three biological replicates) were consigned to Novogene Co., Ltd. (Beijing, China) for total RNA extraction and sequencing. Total RNA was used as input material for RNA sample preparations. RNA integrity was assessed using the RNA Nano 6000 Assay Kit of the Bioanalyzer 2100 system (Agilent Technologies, Santa Clara, CA, USA). After concentration, random disruption, reverse transcription amplification, and purification steps, total RNA that satisfies the integrity test conditions was obtained to yield double-stranded DNA, followed by polymerase chain reaction (PCR) using Phusion high-fidelity DNA polymerase, universal polymerase chain reaction and the exponential (X) primer method. Finally, amplification products were purified (AMPure XP system) and library quality was assessed on an Agilent BioAnalyst 2100 system. The clustering of the index-coded samples was performed on a cBot Cluster Generation System using TruSeq PE Cluster Kit v3-cBot-HS (Illumia) according to the manufacturer’s instructions. After cluster generation, the library preparations were sequenced on an Illumina Novaseq platform.

### 2.5. Quantification of Gene Transcription Level and Analysis of DEGs

Raw data (raw reads) in fastq format were processed through in-house perl scripts, after which clean data (clean reads) were obtained by removing low quality reads and reads containing adapters and N bases from raw data. Then, Q20, Q30, and GC content of clean data were calculated. All the downstream analyses were based on the high-quality clean data. Indexing of the reference genome was performed using HISAT2 v2.0.5, and paired end clean reads were compared to the Citrus Clementine genome. After data quality control, reads mapped to each gene were calculated according to FeatureCounts (1.5.0-p3) [27]. The FPKM (expected number of fragments per kilobase of transcript sequence per million base pairs sequenced) values were then calculated for each gene based on the length of the gene and the number corresponding to that gene was read. Differential expression analysis between the two comparative groups was performed using DESeq2 software (1.20.0) [28]. Benjamini and Hochberg’s method was used to adjust the resulting *p* values to control the false discovery rates (FDRs). Multiple hypothesis testing correction was performed to obtain the FDR value (padj is its common form). The corrected *p* values and |log2FC| were used as thresholds for significant differential expression. Statistical enrichment of DEGs in the KEGG pathway was analyzed using clusterProfiler (3.4.4) software. We used “padj < 0.05 and |log2FC| ≥ 1” as the criterion for determining significant differences in gene expression. Log normalized transcriptome data (Log_2_FPKM + 1) were used for principal component analysis (PCA) and heat map analysis. The online tool Biovenn was used to draw area proportional Venn diagrams [29]. Log_2_FC values from the nine groups were used for MapMan analysis and TBtools mapping. Differences in the expression of genes involved in each functional module in samples from different difference comparison groups were shown with MapMan (version 3.6.0RC1, Berlin, Germany). All genes that could be annotated in regulatory pathways were tagged and their relative expression heat maps were used mainly to show the response of certain metabolic pathways to fruit development and ripening. TBtools was used for heat mapping of DEGs in each module [30].

### 2.6. Quantitative Real-Time PCR (qRT-PCR) Analysis

Total RNA was extracted from fruit pulp tissue at different developmental stages of the three cultivars using RNAprep Pure (Tiangen, Beijing, China). The first-strand cDNA was synthesized using an RNA reverse transcription kit (Toyobo, Shanghai, China). To verify the accuracy of the transcriptome data, we used Primer 5 to design specific primers for 12 genes and performed qRT-PCR analysis (Table S5). These primers were synthesized by Tsingke Biotech, Beijing, China. The qRT-PCR analysis was performed using Bio-Rad CFX Manager (Bio-Rad, Shanghai, China) and SYBR Premix Ex Taq II (novoprotein, Shanghai, China) to validate the DEG expression results. All samples had three biological replicates and were technically replicated three times. Gene expression levels were normalized against the geometric mean of the citrus reference gene β-actin (GenBank: XM_006429010.2) and calculated by the 2^−ΔΔCT^ method [31].

### 2.7. Statistical Analysis

Statistical analysis was performed using SPSS Statistics 19 software (IBM, Armonk, NY, USA). One-way ANOVA and Duncan’s multiple comparison test were used to analyze the differences between samples of the same cultivar at different stages of development and ripening.

## 3. Results

### 3.1. Changes in Fruit Appearance and Physiological Indicators at Different Developmental Stages

**Figure 1:**
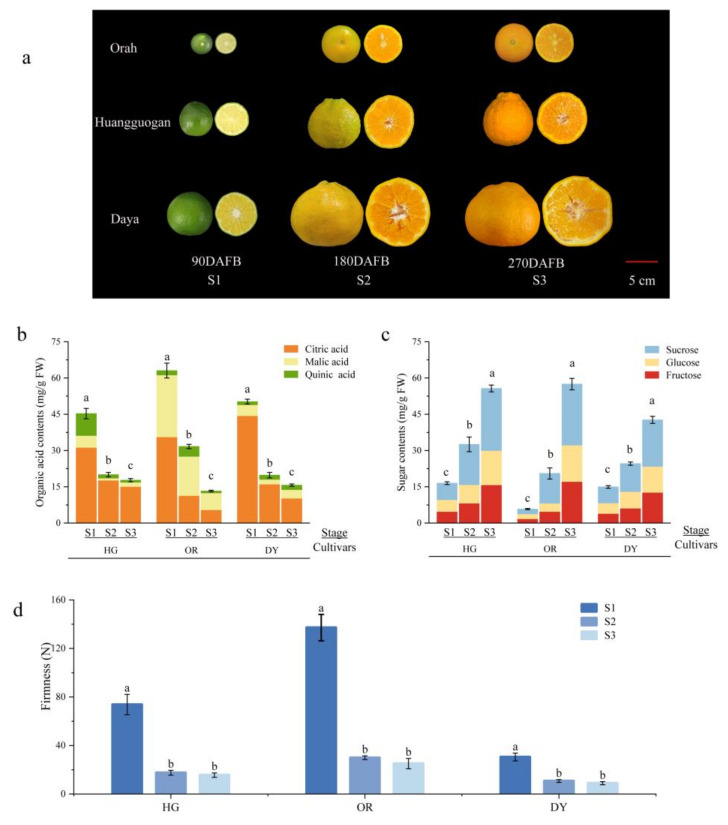
(**a**) Morphological changes during fruit development and ripening of the three cultivars. Changes in organic acid content (**b**) and sugar content (**c**) during fruit development and ripening. Error bars indicate the standard error of total substance content, and different letters indicate significant differences in total content, *p* < 0.05. (**d**) Changes in fruit firmness during fruit development and ripening. S1, stage 1; S2, stage 2; S3, stage 3.

The appearance of the three cultivars is shown in Figure 1a. The fruits undergo cell expansion at stage 1 (S1), form color at stage 2 (S2), and ripen at stage 3 (S3) with a light-orange color of both the peel and flesh. The changes in sugar and organic acid content of the fruits are shown in Figure 1b,c. The organic acid content gradually decreased with fruit development—HG and DY accumulated mainly citric acid, followed by malic acid, while OR had comparable citric and malic acid contents and the lowest total acid content. The sugar content gradually increased with fruit development, with all three cultivars predominantly showing sucrose accumulation. Fruit firmness generally decreased with development and ripening, decreasing significantly from stage 1 to stage 2. Changes in texture occurred mainly during the increase in fruit volume and were not significant after the onset of fruit color change (Figure 1d).

### 3.2. UPLC-MS/MS-Based Quantitative Metabolomic Analysis of the Citrus Fruits

**Figure 2:**
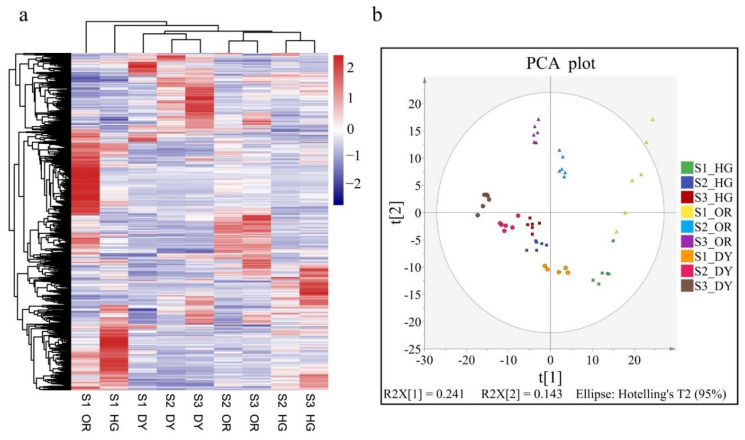
(**a**) Cluster heatmap based on different samples of different metabolites. Red represents up-regulation, and blue represents down-regulation. (**b**) Principal component analysis (PCA) of all samples.

In this study, a total of 1203 metabolites (including 606 in positive mode and 597 in negative mode) were identified, and the differential metabolites were displayed in a clustered heat map (Figure 2a). Samples from S1 of all three cultivars were clearly separated, and samples from S2 and S3 of the same cultivar were clustered together. We performed PCA to visualize the metabolite distribution in all samples (Figure 2b). The PCA score plots showed that all samples were separated from each other. The distribution of samples of each cultivar at S2 and S3 was relatively close, but distant from the samples of S1.

**Figure 3:**
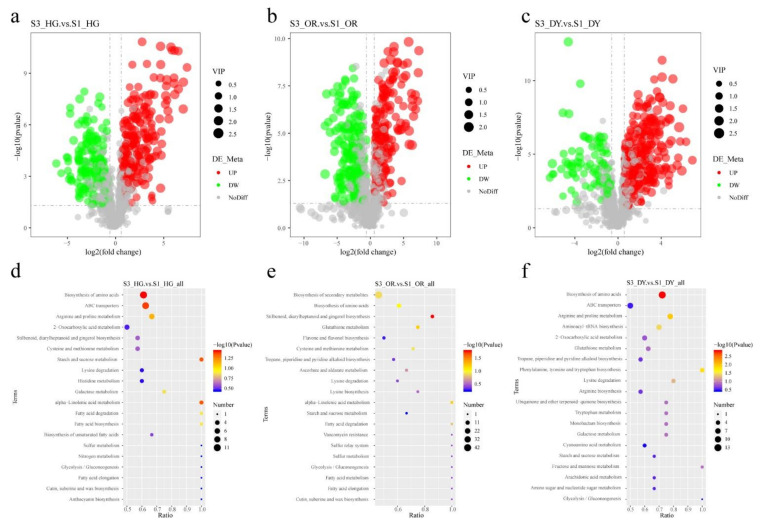
(**a**–**c**) Volcano plot of the metabolic profile at stage 3 (S3) and stage 1 (S1). The red, green and grey circles represent up-regulated, down-regulated, and non-significant metabolites, respectively. The horizontal axis represents the folded change in metabolite content, and the vertical axis represents the level of significance of the difference. Screening criteria are described in the Methods section. (**d**–**f**) KEGG pathway enrichment analysis of differential metabolites at S3 and S1. The horizontal coordinate of the graph is the ratio of the number of differential metabolites annotated to the KEGG pathway to the total number of differential metabolites, and the vertical coordinate is the KEGG pathway. The size of the dots represents the number of metabolites annotated to the KEGG pathway, and the color gradient from red to purple represents the significance of the size of enrichment. Sample 1 vs. Sample 2 represents the different metabolites in the former compared to the latter.

Differential metabolites were detected in samples of the three cultivars at different stages as shown in Figure 3a–c and Figure S1. Compared to S1, 221, 205, and 302 metabolites increased and 172, 222, and 85 metabolites decreased in HG, OR, and DY, respectively, at S3. At S2, 185, 143, and 198 metabolites showed increased levels in HG, OR, and DY, respectively, compared to S1. In contrast, 201, 233, and 124 metabolites were decreased in HG, OR, and DY, respectively. Compared to S2, levels of 198, 156, and 188 metabolites increased and levels of 43, 85, and 37 metabolites decreased at S3 in HG, OR, and DY, respectively. Overall, the number of differential metabolites was greater between S2 and S1 than between S3 and S3.

The differential metabolite details for all comparison groups are presented in Table S2. We mapped these differential metabolites to the KEGG database for enrichment analysis. Based on metabolite enrichment pathways (Figure 3d–f and Figure S2), the differential metabolites between S1 and S3 of the three cultivars were found to be mainly co-enriched in amino acid biosynthesis, glycolysis/gluconeogenesis, lysine degradation, sucrose metabolism, and sulfur metabolism pathways. The differential metabolites between S2 and S1 were mainly co-enriched in arginine and proline metabolism, amino acid biosynthesis, cutin, suberin and wax biosynthesis, cysteine and methionine metabolism, and fatty acid degradation and elongation pathways. The differential metabolites between S3 and S2 of the three cultivars were mainly co-enriched in galactose and sulfur metabolism pathways. This indicates that the enrichment of differential metabolites during the transition from color change to ripening was mainly related to sugar metabolism. The results showed that the metabolic pathways related to sugars, amino acids, fatty acids, and cuticle formation changed significantly with fruit development in all three cultivars and showed similar accumulation patterns.

**Figure 4:**
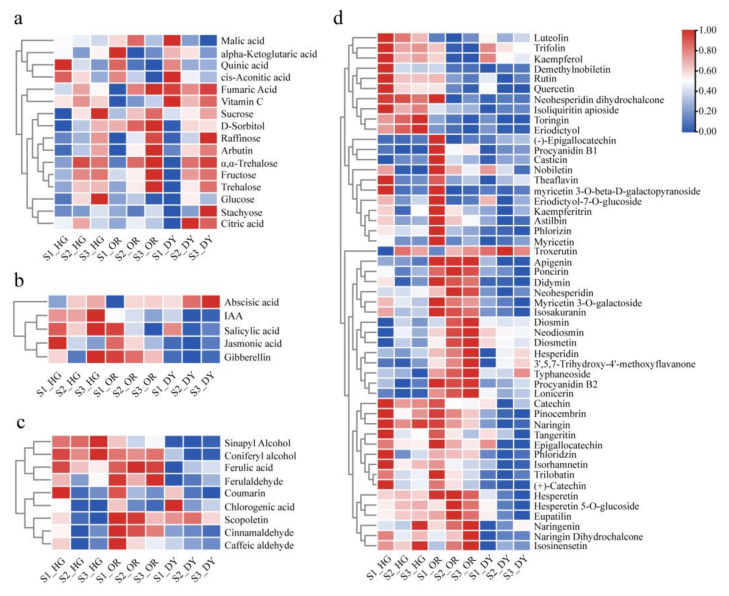
Patterns of accumulation of metabolites involved in (**a**) sugars and organic acids, (**b**) plant hormones, (**c**) lignin synthesis, and (**d**) flavonoid biosynthetic pathways. In the heat map, the rows represent metabolites and the columns represent samples. Colors indicate the level of metabolite accumulation in different samples. The gradation in color (from blue to white to red) indicates low to high levels of metabolites.

As previously reported, primary and secondary metabolites such as sugars, organic acids, and flavonoids are important components that determine flavor and nutritional quality of citrus [32,33]. In addition, the texture of citrus pulp is an important quality trait and determinant of maturation, and many studies have shown that lignin also affects the firmness and masticatory properties of plants and fruits [34,35]. We explored the fruit quality-related metabolites in the three cultivars, including sugars, acids, citrulline, flavonoids, lignin synthesis precursors, and hormones that regulate fruit development; their accumulation patterns at different developmental stages are shown in Figure 4. The content of carbohydrates (such as fructose, sucrose, and glucose), limonin, and vitamin C gradually increased with fruit development, while the content of organic acids, such as citric acid, malic acid, and quinic acid gradually decreased (Figure 4a). The levels of three sugars and three organic acids were determined by high performance liquid chromatography (HPLC) and the results were found to be consistent with our metabolomic analysis, indicating that the metabolomic data are reliable (Figure 4b,c). In addition, five hormones that regulate fruit development were identified (b). The abscisic acid content in all three cultivars were the lowest at S1 and gradually increased with the fruit development and ripening. In contrast, jasmonic acid content was the highest at S1 and gradually decreased with fruit development and ripening. Variety-specific changes were observed for gibberellin and indole-3-acetic acid (IAA). The trend of salicylic acid accumulation was opposite to that of abscisic acid, with high content in the early stages of development that decreased as the fruit development and ripening progressed. Nine metabolites of the phenylpropanoid/lignin synthesis pathway were identified (sinapyl alcohol, coniferyl alcohol, ferulic acid, scopoletin, ferulaldehyde, cinnamaldehyde, coumarin, caffeic aldehyde, and chlorogenic acid), and most of them showed high accumulation during early developmental stages (Figure 4c). A total of 51 flavonoid metabolites were identified, most of which showed high accumulation at S1 and then gradually decreased with fruit development (Figure 4d). The levels of the most important citrus flavonoids such as tangeritin, rutin, and naringin peaked during the early stages of development followed by a gradual decrease, whereas the levels of naringenin and hesperidin gradually increased with fruit development. In addition, the hesperetin content did not vary significantly throughout the different stages.

### 3.3. General Description of the Transcriptome

At the stages involving fruit-expansion, color change, and ripening, the three cultivars of citrus were divided into nine groups, and each group included three replicates. After data quality control, we obtained 179.39 Gb of clean bases, with 44,290,704 average number of clean reads. The average mapping rate of these reads after comparing them with the genome was 92.68%. Approximately 173.39 GB data of clean reads were obtained with a Q30 ≥ 92.7% and GC content between 43.33 and 44.69%; the data was considered to be highly reliable for further study. These data are presented in Table S3.

**Figure 5:**
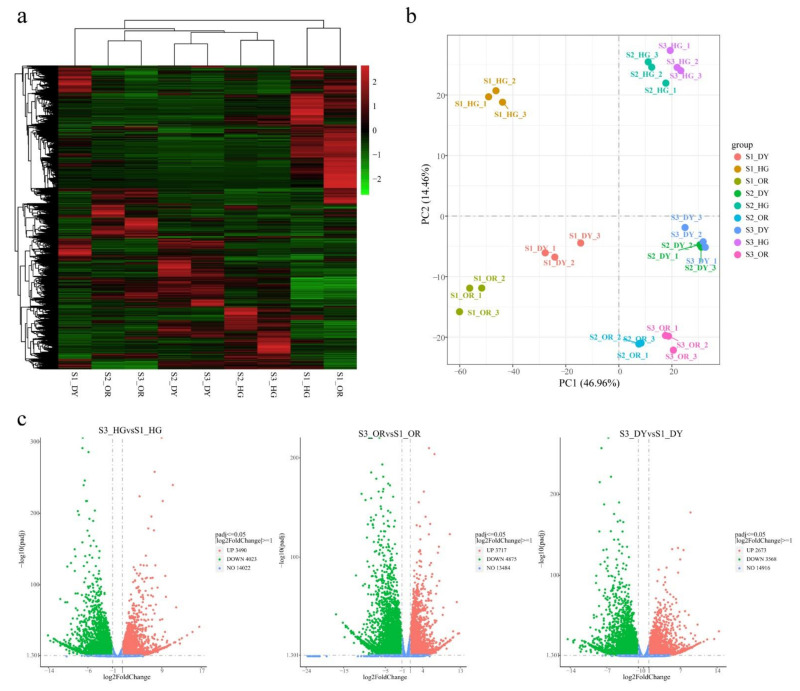
(**a**) Cluster heat map of differentially expressed genes (DEGs) between different samples. Each column in the graph represents a sample, and each row represents a gene. The color in the graph indicates the amount of gene expression in the sample (log10(FPKM+1)). Red color indicates that the gene is highly expressed in the sample, and green color indicates that the gene is lowly expressed. (**b**) PCA scatter plots of different samples based on transcriptome data. (**c**) Volcano plots of DEGs across different groups. The red dots, inns, and blue dots represent up-regulated, down-regulated, and non-significant DEGs, respectively. The horizontal axis represents fold change in gene expression levels, and the vertical axis represents the significance of differences.

In this study, we calculated fragments per kilobase of transcript per million base pairs (FPKM) for individual genes in all samples to calibrate expression values and compared gene expression differences at different developmental stages for each hybrid citrus cultivar. Systematic cluster analysis of transcript abundance was performed for all samples based on transcriptome data. Expression data of DEGs were used for cluster heat map analysis (Figure 5a). The samples were divided into two groups, one group including S1 and the other including S2 and S3 samples, which were separated from each other and differed significantly between cultivars. The PCA score plot showed the differences in transcriptome between the samples (Figure 5b). The samples of S1 were more discrete at PC1 and PC2 than the samples of S2 and S3, and the samples of S1 were distributed on the negative half-axis of PC1, away from the samples of S2 and S3. The difference between the samples of S2 and S3 was lesser than their individual differences with S1, which is consistent with the cluster heat map results.

We used “padj < 0.05 & |log_2_FoldChange| ≥ 1” as the criterion for significant differences in gene expression. DEGs were considered to be up-regulated when log_2_ FoldChange > 1 and down-regulated when log_2_FoldChange < −1. Volcano plots showed the number of DEGs between each of the analyzed combinations. We analyzed the up- and down-regulation of DEGs at different developmental stages in the three cultivars, and the results are shown in Figure 5c and Figure S3. The number of DEGs was greater for the comparative group “S2 vs. S1” than for “S3 vs. S2”, and “S3 vs. S1” group had the highest number of DEGs. This result is similar to the results of the cluster heat map and PCA.

We mapped DEGs to reference pathways in the KEGG database and identified the biological pathways in which these genes may be involved; Figure S4 shows the KEGG metabolic pathways enriched for DEGs between different developmental stages of each cultivar. Two metabolic pathways were significantly enriched in all the nine groups, including plant hormone signal transduction and phenylpropanoid biosynthesis. Two metabolic pathways—flavonoid biosynthesis and photosynthesis—antenna proteins—were found to be significantly enriched in six groups. In summary, the highest enrichment was found in the phenylpropanoid biosynthesis and the plant hormone signaling pathways, which were present throughout the growth and maturation of all cultivars, followed by the flavonoid biosynthesis and photosynthesis—antenna proteins pathways.

**Figure 6:**
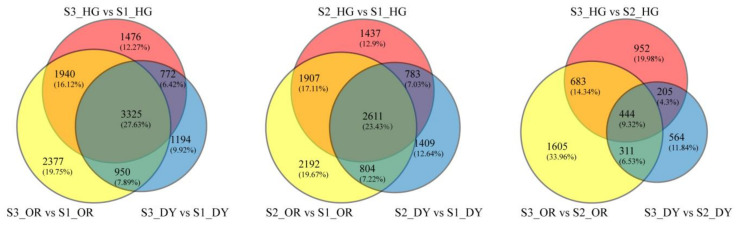
Venn diagrams of differentially expressed genes in the studied groups at different developmental stages of each cultivar. The number outside the parentheses is the number of genes contained in each region. The percentage of each region’s area to the total area is in parentheses.

The Venn diagrams show the overlap of all DEGs present in each developmental stage comparison group of the three cultivars (Figure 6). The DEGs in the central overlap area are common to all cultivars, which is specifically of interest. There are 3325 DEGs common to all three cultivars between stage 3 and stage 1, 2611 DEGs common between stage 2 and stage 1, and 444 DEGs common between stage 3 and stage 2. All genes in the central overlap of the three Venn diagrams (3689 genes in total) were mapped to the software Mapman’s Clementine citrus database for gene annotation and functional classification. Gene expression levels were mapped using log_2_FC values of DEGs between the two samples. Multiple overview maps of the pathways of these genes in the nine comparison groups were also obtained. We focused on four pathway overviews, namely “metabolic overview”, “secondary metabolism”, “regulatory overview”, and “regulation”.

### 3.4. Differentially Expressed Endogenous Metabolism-Related Genes at Different Developmental Stages

**Figure 7:**
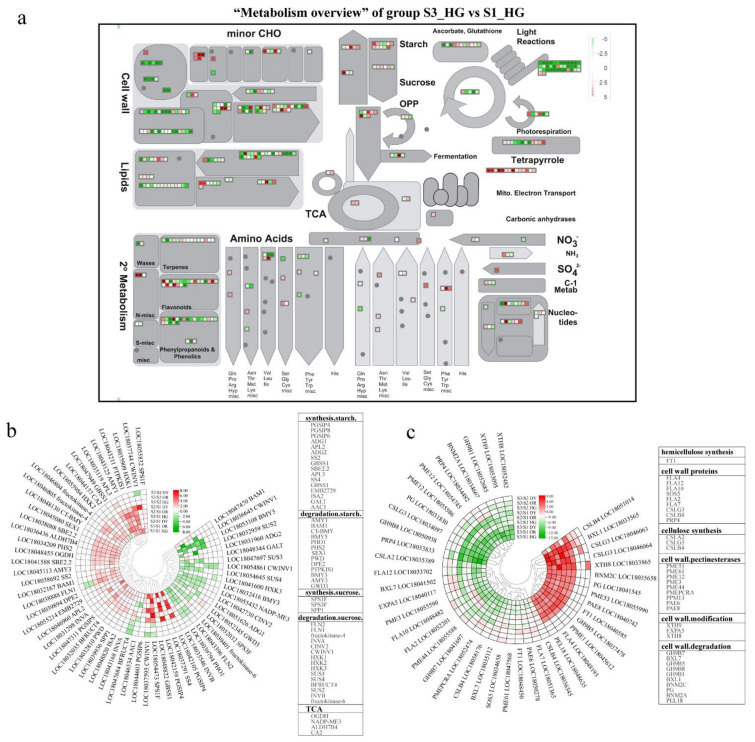
(**a**) MapMan-based “metabolic overview map” of DEGs in the “S3_HG vs. S1_HG” group, including lipid, cell wall, secondary metabolism, starch, sucrose, tricarboxylic acid (TCA) cycle, amino acid, and light reaction components. The small heat map in each section of the diagram shows the DEGs mapped to that pathway, and a small square indicates a gene. The color of the square indicates the expression of the gene at S3, with green and red indicating down-regulation and up-regulation, respectively. (**b**) Differentially expressed genes (DEGs) related to starch, sucrose, and TCA metabolism during tangor fruit development and ripening. (**c**) DEGs related to cell wall metabolism during tangor fruit development and ripening. Heatmap of DEGs was drawn using the log_2_Foldchange value obtained from the pairwise comparison of samples. S3/S1, comparison group of S3 and S1; S2/S1, comparison group of S2 and S1; S3/S2, comparison group of S3 and S2. Red and green indicate up-regulation and down-regulation, respectively, in the former of the comparisons.

To understand the changes in transcript levels of metabolic pathways during tangor fruit development and to further investigate the key DEGs, we obtained “metabolic overview” profiles of nine comparison groups using Mapman (Figure 7a and Figure S5). The results showed that functional modules such as lipids, cell wall, secondary metabolism, starch, sucrose, tricarboxylic acid (TCA) cycle, amino acids, and light reactions are regulated to varying degrees during fruit development and ripening. DEGs that were common to all three cultivars had more down-regulated genes than up-regulated genes as the fruit developed and ripened. These genes were more differentially expressed in the early stages of development (stage 1 to stage 2), and the number of DEGs became significantly less during the process of fruit color change to ripening. Here, we focused on DEGs related to sucrose and starch metabolism, TCA cycle, and cell wall metabolism.

Cluster analysis of DEGs in the functional modules of starch, sucrose metabolism, and TCA cycle was performed. A total of 61 DEGs were found to be associated with starch, sucrose metabolism, and TCA cycle for the nine groups; the information about these genes is presented in the heat map (Figure 7b). Among the DEGs associated with starch and sucrose metabolism, the sucrose degradation-related gene *HXK1* (LOC18035909) and the starch degradation-related genes *PTPKIS1* (LOC18043211) and *AMY1* (LOC18043125) were up-regulated throughout development and ripening, and the starch degradation-related gene *BMY5* (LOC18053108) was down-regulated throughout fruit development. Three of the DEGs associated with the TCA cycle [*OGDH* (LOC18048455), *ALDH7B4* (LOC18036436), and *CA2* (LOC18044137)] were up-regulated throughout development and ripening in all cultivars. In contrast, only one gene, *NADP-ME3* (LOC18055432), was down-regulated throughout development and ripening.

The DEGs related to cell wall metabolism are shown in Figure 7c. There were mainly 44 DEGs in all samples, of which 31 genes were down-regulated throughout fruit development and ripening, and 13 genes were up-regulated at both S2 and S3. However, no significant difference was found in the expression levels of these up-regulated genes between S2 and S3, suggesting that the most active period of pulp cell wall metabolism is from S1 to S2. The up-regulated genes included *PG* (LOC18041545) and *PME53* (LOC18055990), both of which have been shown to play important roles in sweet orange cell wall metabolism in previous studies [36]. Additionally, one of the up-regulated genes, *BXL1* (LOC18033565), which is involved in cell wall degradation, was significantly more up-regulated in the S2 and S3 samples than the other genes. Among the down-regulated genes, *PME44* (LOC18055588), *FLA2* (LOC18052201), and *FLA10* (LOC18048482) were down-regulated in all nine groups.

**Figure 8:**
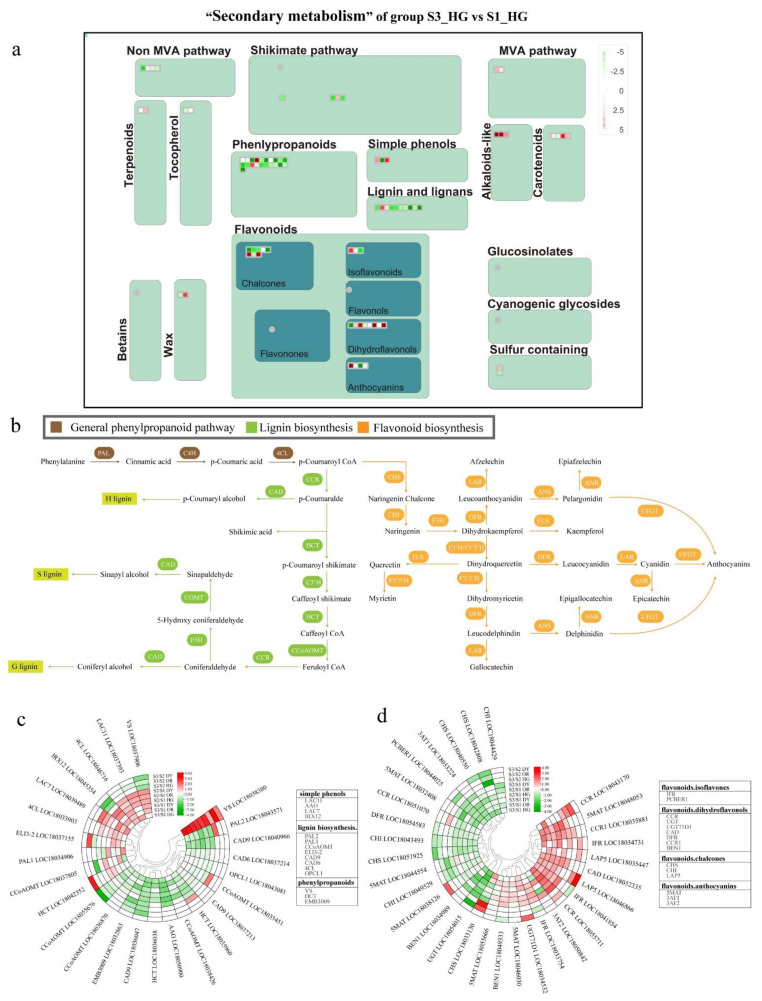
(**a**) MapMan-based “metabolic overview map” of DEGs in the “S3_HG vs. S1_HG” group, including modules for phenyl propane, lignans, flavonoids, mangiferylic acid, and wax. The small heat map in each section of the figure shows the DEGs mapped to that pathway, with a small square indicating a gene. The color of the square indicates the expression of that gene in the third stage, down-regulated in green and up-regulated in red. (**b**) General phenylpropanoid metabolism, lignin biosynthesis, and flavonoid biosynthesis metabolic pathways in citrus. Differentially expressed genes associated with (**c**) monophenol, phenylpropanoid, and lignin biosynthesis, and (**d**) flavonoid biosynthesis metabolism during fruit development and ripening. Red indicates up-regulation, and green indicates down-regulation. Heatmap of DEGs was drawn using the log_2_Foldchange value obtained from the pairwise comparison of samples. S3/S1, comparison group of S3 and S1; S2/S1, comparison group of S2 and S1; S3/S2, comparison group of S3 and S2. Red and green indicate up-regulation and down-regulation, respectively, in the former of the comparisons.

To understand the changes in transcript levels of secondary metabolic pathways during tangor fruit development and ripening and to further explore the key DEGs, we obtained an overview map of “secondary metabolism” (Figure 8a and Figure S6). As fruit development and ripening progressed, genes associated with biosynthetic pathways such as that of phenylpropanoids, simple phenols, lignin and lignans, flavonoids, and carotenoids were differentially expressed. As shown in Figure S5, the number of DEGs was greater between S2 and S1 than between S3 and S2, and the transcript levels of fruit secondary metabolic pathways were more variable in the early stages of development. The DEGs mapped to the overview map were mainly concentrated in phenyl propane, lignin, and flavonoids, which were our focus modules.

The branches of phenylpropanoid metabolism produce end products including flavonoids, hydroxycinnamic acid esters, hydroxycinnamic acid amides (HCAAs), and precursors of lignin, lignans, and tannins, the most prominent of which are lignin, lignans, and flavonoids [37]. We analyzed the phenylpropanoid/lignin synthesis and flavonoid metabolic pathways and the associated DEGs (Figure 8b–d). For phenylpropane, lignin biosynthesis, and monophenol metabolism, 26 major DEGs were identified in the nine groups (Figure 8c). A phenylpropane-related gene *VS* (*vinorine synthase*, LOC18038380) and a lignin biosynthesis-related gene 4CL were up-regulated throughout fruit development and maturation. In contrast, seven genes were generally down-regulated throughout fruit development and ripening—two *CAD9* (LOC18036047, LOC18040966), *PAL2* (LOC18043571), *CCoAOMT* (LOC18055676), *CAD6* (LOC18037214), *HCT* (LOC18035960), and *EMB3009* (LOC18032863). Additionally, a phenylpropane related gene *VS* (LOC18037906) was up-regulated during S1 to S2 and down-regulated during S2 to S3. For the flavonoid metabolic pathway, 31 DEGs were present in the nine groups (Figure 8d). Compared to S1, five genes were up-regulated at S2 and S3, namely *IFR* (LOC18041854), *CAD* (LOC18052335), *CCR1* (LOC18035881), *CCR* (LOC18055711), and *5MAT* (LOC18048053). Conversely, five genes were down-regulated, namely *CHS* (LOC18051925), *CHI* (LOC18043493), *PCBER1* (LOC18044025), *UGT* (LOC18054015), and *DFR* (LOC18054583).

To verify the accuracy and reproducibility of the transcriptome analysis results, 12 flavonoid structural genes were randomly selected for qRT-PCR validation (Figure S7). qRT-PCR analysis showed the same expression trends as the RNA-Seq data for all 12 genes. Hence, the reliability of the RNA-Seq data was confirmed.

### 3.5. Plant Hormone-Related DEGs during Fruit Development and Ripening

**Figure 9:**
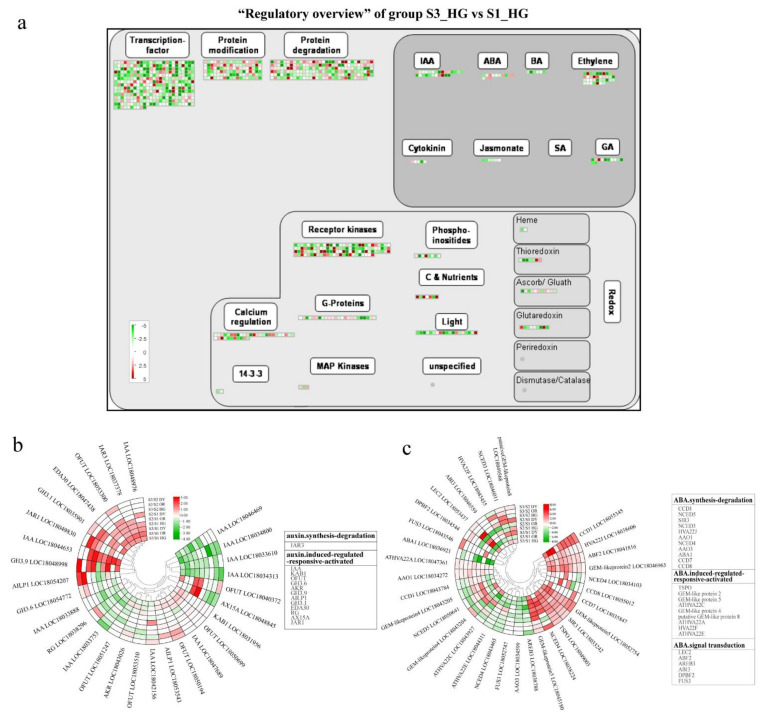
(**a**) MapMan-based “regulatory overview map” of the “S3_HG vs. S1_HG” group of DEGs, including transcription-factors, phytohormones, protein modification, protein degradation, receptor kinases, calcium regulation, G-protein, and C & Nutrients modules. The small heat map in each section of the figure shows the DEGs mapped to the pathway, with a small square indicating a gene. The color of the square indicates the expression of that gene at stage 3, with green showing down-regulation and red showing up-regulation. Plant hormone-related differentially expressed genes during development and ripening in tangor citrus: (**b**) auxin; (**c**) abscisic acid; red and green represent up- and down-regulation, respectively. Heatmap of DEGs was drawn using the log_2_Foldchange value obtained from the pairwise comparison of samples. S3/S1, comparison group of S3 and S1; S2/S1, comparison group of S2 and S1; S3/S2, comparison group of S3 and S2. Red and green indicate up-regulation and down-regulation, respectively, in the former of the comparisons.

To understand the tangor fruit transcriptional regulatory processes in response to fruit development and ripening, we mapped DEGs to a “regulatory overview” graph in MapMan. The color distribution of the heat map shows that the expression of genes related to transcription-factor, phytohormones, protein modification, protein degradation, receptor kinases, calcium regulation, G-protein, and C & Nutrients and other related genes are regulated by the fruit development and ripening process (Figure 9a and Figure S8). In these modules, the number of DEGs during the development from S1 to S2 is much higher than during S2 to S3. In the hormone biosynthesis pathway, some genes involved in the synthesis of growth hormone, abscisic acid, ethylene, gibberellin, and jasmonic acid were expressed during the growth and development of tangor fruits.

Plant hormones are important in the regulation of fruit development and ripening. Abscisic acid was considered to be a ripening control factor for non-leaping fruits, which promotes ripening in citrus fruits and plays an important regulatory role during fruit development [19,38]. There were 32 abscisic acid-related DEGs involved in abscisic acid synthesis, degradation, signal transduction, and activation of induced regulatory responses in the nine groups (Figure 9b). Five of these genes are commonly up-regulated during fruit development and ripening, including a signal transduction gene *ABF2*, two synthesis and degradation related genes (*SIR* and *CCD1*), and two genes that induce activation of regulatory responses (*TSPO* and *GEM-like protein 5*). However, one inducible gene, *ATHVA22E* (LOC 18044311), two synthesis and degradation related genes, *AAO* (LOC 18034599) and *CCD1* (LOC 18043784), and one signal transduction gene, *AREB3* (LOC 18038788), were down-regulated at both S2 and S3.

Previous studies have shown that auxin plays an important regulatory role in fruit set, growth, and ripening [39]. The expression of 28 auxin-related DEGs in the nine groups, which were mainly involved in auxin-induced regulation of response activation, is shown in Figure 9c. Of these, four genes were up-regulated throughout fruit development and ripening, namely two *IAA* genes (LOC18048976 and LOC18044653, one *IAR3* gene (LOC18037378), and one *EDA30* gene (LOC18047438). Two *IAA* genes (LOC18034800 and LOC18033753) were down-regulated during fruit development and ripening. Three genes were down-regulated from S1 to S2 and up-regulated from S2 to S3, namely two *IAA* genes (LOC18046469 and LOC18033888) and one *OFUT* gene (LOC18051247). Although their expression levels were upregulated at S3 relative to S2, the levels remained lower than that at S1 after ripening (S3).

The expression profiles of the other four hormone-related DEGs also showed some developmental stage-specificity (Figure S9). More than half of the ethylene-related genes in all varieties were expressed at lower levels at S3 than at S1, with an overall down-regulated expression throughout fruit development and ripening. The expression levels of genes related to ethylene synthesis and degradation (*SRG*, *GA20OX2*, and *F6′H1*) and signal transduction genes (*ERF3*) were higher at S3 than at S1. In addition, three signal transduction-related ethylene-responsive transcription factors (*ERF4*, *ERF1*, *ERF6*), a biosynthetic gene (*ACO1*), and an inducible gene (*PHOS34*) were down-regulated in the S2/S1 group and up-regulated in S3/S2. Expression of the cytokinin-related gene *CKX1* was up-regulated throughout fruit development and ripening. In contrast, *CKI1* and *HK1* were mainly highly expressed at early stages of development (S1) and down-regulated thereafter. Among the DEGs for gibberellic acid, a synthetic degradation gene (*GA20OX1*) and an induced regulatory response activation gene (*GASA14*) were up- and down-regulated, respectively, throughout fruit development and ripening.

### 3.6. Differential Expression of Transcriptional Regulatory Genes during Fruit Development and Ripening

**Figure 10:**
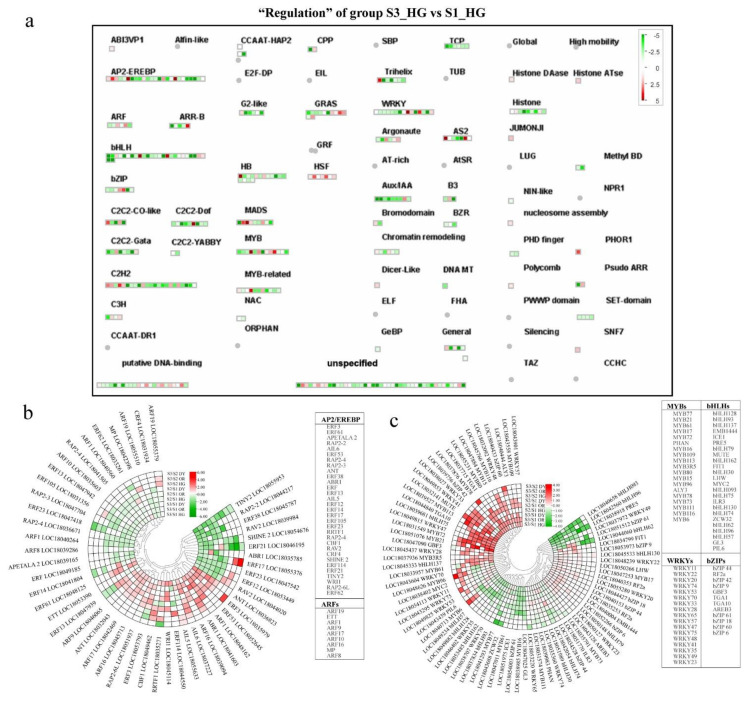
(**a**) The MapMan-based “S3_HG vs. S1_HG” DEG group transcription factor overview “Regulation” contains various transcription factor modules such as AP2, ARF, bHLH, bZIP, WRKY, and MYB. The small heat map in each section of the figure shows the DEGs mapped to the transcription factor modules, and the small squares indicate the genes. The color of the square indicates the expression of the gene at stage 3, with green indicating downregulation and red indicating upregulation. The heat map shows the relative transcript levels of various transcription factor genes between the two developmental stages. Circular heat map shows differentially expressed genes in the (**b**) AP2/EREBP and ARF transcription factor families, and (**c**) MYBs, WRKYs, bHLHs, and bZIPs transcription factor families during tangor fruit development and ripening. Heatmap of DEGs was drawn using the log_2_Foldchange value obtained from the pairwise comparison of samples. S3/S1, comparison group of S3 and S1; S2/S1, comparison group of S2 and S1; S3/S2, comparison group of S3 and S2. Red and green indicate up-regulation and down-regulation, respectively, in the former of the comparisons.

As seen in the Regulatory Overview, the transcription factor module maps the most DEGs, with more than half of the transcription factor genes being downregulated at S3 compared to S1. Transcription factors play an important regulatory role in tangor fruit development. To further investigate the response of transcription factors to fruit development and their specific expression patterns, we specifically mapped DEGs to the transcription factor overview “regulation” (Figure 10a and Figure S10).

We analyzed several major transcription factor families that are present in plants. AP2-EREBP (APETALA2 and ethylene-responsive element binding proteins) is extensively involved in plant biological responses, regulates various plant developmental processes, and plays an important role in hormone regulation and stress response [40,41]. Two DEGs of the AP2-EREBP transcription factor family—*AIL6* (LOC18037227) and *ANT* (LOC18036823)—were up-regulated throughout the development in all three cultivars (Figure 10b). Members of the auxin response factor (*ARF*) family are thought to play a key role in regulating fruit development and the expression of auxin response genes [42]. The ARF transcription factor family genes *ARF17* (LOC18042469) and *ARF1* (LOC18048162) were up-regulated throughout fruit development in all three cultivars (Figure 10b). Many MYB proteins are involved in the control of primary and secondary metabolism and developmental processes in plants [43]. More number of MYB transcription factor family genes were up-regulated at S3 than other stages, with three genes up-regulated in all three varieties, *MYB72*, *MYB109* and *MYB80*. In contrast, four MYB genes were simultaneously down-regulated throughout fruit development and ripening in all three cultivars (Figure 10c). In the WRKY transcription factor family, only *WRKY 47* was up-regulated during the fruit development and ripening process of the three cultivars. *WRKY 48* gene was simultaneously up-regulated at S2 of all three cultivars (Figure 10c). Most genes of the bHLH and bZIP transcription factor families were down-regulated throughout the development, and only four genes from these transcription factor families were up-regulated at both S2 and S3 (*bHLH 137* and *bHLH 75*, *TGA1* and *TGA10*) (Figure 10c).

## 4. Discussion

The molecular basis of fruit quality or growth and development in citrus fruits of sweet orange, grapefruit, and mandarin types has been studied previously; however, reports on mandarin-orange hybrids are scarce. In this study, metabolomic and transcriptomic analyses were conducted for the first time on fruits of mandarin-orange hybrids, one of the citrus hybrids, at different developmental stages to comprehensively reveal the accumulation patterns of primary and secondary metabolites as well as the gene expression patterns of endogenous metabolism, hormones, and transcriptional regulation during tangor fruit development and to better understand the molecular mechanisms of tangor fruit quality formation and fruit development and ripening.

Fruit quality has been a bottleneck to the development of the citrus industry. Researchers have been exploring methods to improve fruit quality, particularly to improve the nutritional quality of citrus pulp, masticatory traits, total soluble solids, and titratable acids (TA). Primary and secondary metabolites such as sugars, organic acids, and flavonoids are important for the flavor and nutritional quality of citrus [32,33]. The development and ripening process of tangor fruits is accompanied by the accumulation of sugars (mainly sucrose) and degradation of acids, such as citric acid (Figure 1b–c and Figure 4a), which is consistent with previous studies on citrus sugars and organic acids [44]. Starch and sucrose metabolism contribute to the development of sweetness in citrus fruits during ripening, whereas citric acid is the most important factor in constituting the sourness of the fruit, whose accumulation is mainly catalyzed by the TCA cycle [45]. In this study, few genes were identified with expression patterns consistent with the accumulation patterns of sugars and organic acids, such as the starch degradation-related genes *PTPKIS 1* and *AMY 1*, *OGDH* in the TCA cycle, and *CA2* (Figure 7a, Table S4). These may be the key genes that play a critical role in sucrose and citric acid accumulation. Flavonoids are the most important bioactive substances and antioxidants in citrus, which are also responsible for the bitterness in the fruits [7,46]. In this study, 51 flavonoid metabolites were detected, including tangeritin, rutin, naringin, naringenin, hesperidin, and hesperetin (Figure 4d), which are the major flavonoids in citrus [47]. The content of these metabolites was found to vary during development. The early stages of fruit development show peak accumulation of most flavonoid metabolites; additionally, during early stages, more antioxidants can be extracted from the young fruits of citrus hybrids, which are a better source of bioactive components. We identified several genes of the flavonoid biosynthetic pathway that were up- and down-regulated during fruit development, such as *CCR1*, *IFR*, *DFR*, and *CHI* (Figure 8d, Table S4).

The firmness of citrus fruits is an important quality trait and determinant of ripening, which depends on the cell wall metabolism [8]. Polygalacturonase (*PG*) and pectin methyl esterase (*PME*) in pectin metabolism and genes related to lignin biosynthesis (such as *PALS*, *C4Hs*, *4CLs*, *CCoAOMTs*, and *CADs*) play an important role in citrus cell wall metabolism [36]. In this study, many genes involved in cell wall metabolism were differentially expressed, particularly from S1 to S2. *PME* has been reported to be involved in early cell wall breakdown and also synergistically with *PG*s in the degradation of protopectin, which is a prerequisite for PGs to participate in fruit ripening [48]. We identified one *PG* gene and one *PME53* gene that were significantly up-regulated at both S2 and S3 and negatively correlated with changes in fruit firmness (Figure 7c, Table S4). In addition, numerous studies have shown that firmness and masticatory properties of plants and fruits are influenced by the lignin content [34,35]. In citrus fruits, the accumulation of lignin reduces the water content of the juice sacs, which in turn leads to granulation of the fruit, resulting in poor mastication characteristics [49]. In this study, nine lignin synthesis precursors were initially identified by metabolomic analysis, including two direct precursors—coniferyl alcohol and sinapyl alcohol (Figure 4c). These lignin synthesis precursors were highly accumulated mainly at S1. This may be related to the high firmness in the early stages of fruit development. Many DEGs in the lignin synthesis pathway were identified using transcriptome sequencing, including several genes that were down-regulated during fruit development, such as *CAD9*, *CCoAOMT*, *CAD6*, and *HCT* (Figure 8b, Table S4). The expression patterns of these genes were consistent with changes in the content of precursors of lignin synthesis, and they may play a critical role in lignin synthesis.

Fruit development and ripening is the result of a complex interactions of molecular, biochemical, and physiological processes that are regulated by endogenous factors, such as hormones, and exogenous factors, such as the environment. In this study, only abscisic acid, which is a key hormone that regulates tangor development and ripening, increased gradually with the progression of fruit development and ripening in the fruits of all three cultivars (Figure 4b). Several genes related to abscisic acid synthesis and signal transduction were differentially expressed during development (Figure 9b). The expression levels of the abscisic acid signal transduction gene *ABF2* followed the same trend as the abscisic acid content, which was similar to the results of the study on sweet orange [19]. *ABF2* may be a critical gene that plays a role in abscisic acid signaling during tangor citrus fruit development and ripening. The content of jasmonic acid and salicylic acid decreased gradually with fruit development and ripening. Some key jasmonic acid-related genes were identified, such as *LOX5* (LOC18031493) and *LOX2* (LOC18052054). The expression patterns of these genes were consistent with their accumulation patterns, with their expression being down-regulated as the fruit development progressed (Figure 4b and Figure S9b, and Table S4). However, no salicylic acid-related DEGs were found, and the response of salicylic acid to fruit growth and development may not be obvious. Metabolomic analysis results showed that the accumulation pattern of auxin (IAA) at the three stages was ambiguous (Figure 4b), but transcriptomic analysis revealed that some auxin-related genes were differentially expressed at different stages of fruit development and ripening, such as *IAA*, *IAR3*, and OFUT (Figure 9b, Table S4). Furthermore, some auxin-response-factor transcription factor family genes were also found to be differentially expressed during fruit ripening and development, such as *ARF17* and *ARF1* (Figure 10b, Table S4). ARF genes are expressed in dynamic and differential patterning during development, and genetic studies have shown that individual ARFs control distinct developmental processes [50]. These genes may be involved in the regulation of tangor fruit development and ripening. DEGs of other plant hormones have no obvious expression trend during fruit development and ripening, and they may have more complex mechanisms of action.

Fruit growth and development, metabolite biosynthesis, and hormone signal transduction induce the expression of multiple genes mediated by transcription factors in plants, including AP2/EREBP, MYB, WRKY, bZIP, and bHLH [51]. In this study, many *MYB* transcription factor genes were found to be differentially expressed during fruit development, but only three genes—*MYB72*, *MYB80*, and *MYB109*—were consistently up-regulated during fruit development and ripening (Figure 10b, Table S4). Studies on the function of these genes in plant development have not yet been reported yet, and their role in fruit development needs to be further studied. Moreover, *WRKY47* gene was found to be significantly up-regulated during fruit development and ripening (Figure 10b, Table S4). In *Arabidopsis thaliana, WRKY47* transcription factor regulates the expression of the cell wall modification genes, *XTH17* and *ELP*, as reported in a previous study [52]. *WRKY47* may also be associated with cell wall metabolism in citrus hybrids and plays a role in fruit texture formation; its specific mechanism of action needs to be further investigated. In addition, many genes of the bHLH and bZIP transcription factor families were also found to be up- or down-regulated during growth and development, and these transcription factors may play an important role in fruit development (Figure 10b, Table S4). The specific roles of these transcription factors in tangor fruit development and quality formation need to be further studied.

## 5. Conclusions

Metabolomic and transcriptomic analyses at different developmental stages of the fruit pulp of tangor, one of the mandarin hybrids, were conducted. Metabolite and gene expression analyses indicated that metabolites and genes related to sucrose, starch, cell wall, hormone, phenylpropanoid, and flavonoid metabolism play important roles in fruit development and ripening. Abscisic acid is the most critical plant hormone that regulates the development and ripening of tangor fruits. In addition, many key genes that play a critical role in these metabolic pathways and transcription factors that may be involved in regulating fruit development and ripening were identified. However, the functions of these genes and the mechanism of action of transcription factors have not been clarified and can be the topic of future research. Moreover, the function and interactions between these genes during fruit development can be further studied. In conclusion, the results of this study can provide basic data and theoretical basis for further research on the mechanism of fruit development and quality formation in citrus hybrids.

## Data Availability

Transcriptome data are available at the National Center for Biotechnology Information (NCBI) database under the accession number PRJNA815885 (https://dataview.ncbi.nlm.nih.gov/object/PRJNA815885, accessed on 19 March 2022). Metabolomic data are available in the metabolight database under project number MTBLS4630.

## Supplementary Materials

The following supporting information can be downloaded at: https://www.mdpi.com/article/10.3390/ijms23105457/s1.
