# Supplementary material for: Combined Transcriptome and Metabolome Analyses Reveal Candidate Genes Involved in Tangor (Citrus reticulata × Citrus sinensis) Fruit Development and Quality Formation
Source: Int J Mol Sci. 2022 May 13;23(10):5457. doi: 10.3390/ijms23105457 (PMC9141862; doi:10.3390/ijms23105457)
Supplement: Supplementary file 1 [file ijms-23-05457-s001.zip › Supplementary figures.pdf]

International Journal of
Molecular Sciences

Supplementary figures

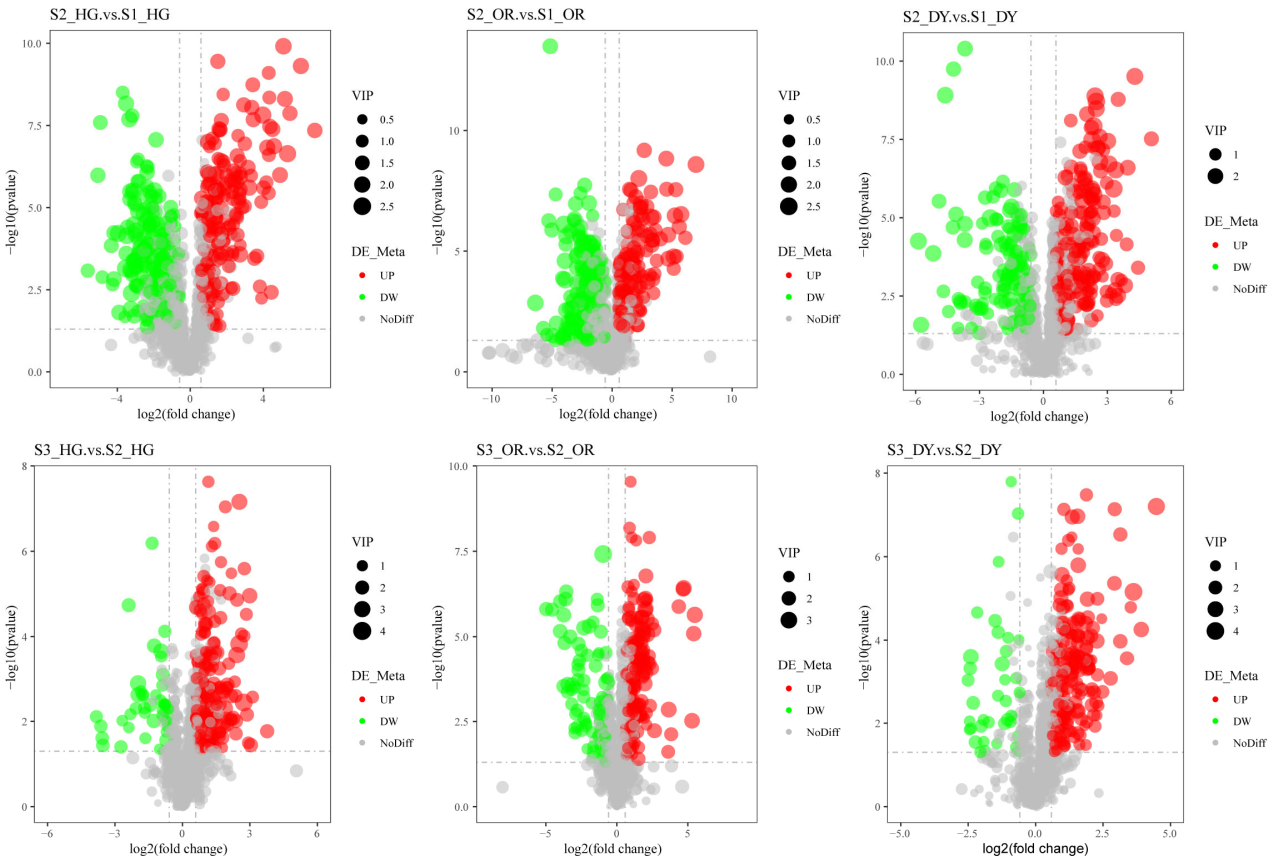

Figure S1. Volcano plots of differential metabolites for comparison groups S2vsS1 and S3vsS1 for the three cultivars. The red, green and grey circles represent up-regulated, down-regulated, and non-significant metabolites, respectively. The horizontal axis represents the folded change in metabolite content, and the vertical axis represents the level of significance of the difference. Screening criteria are described in the Methods section.

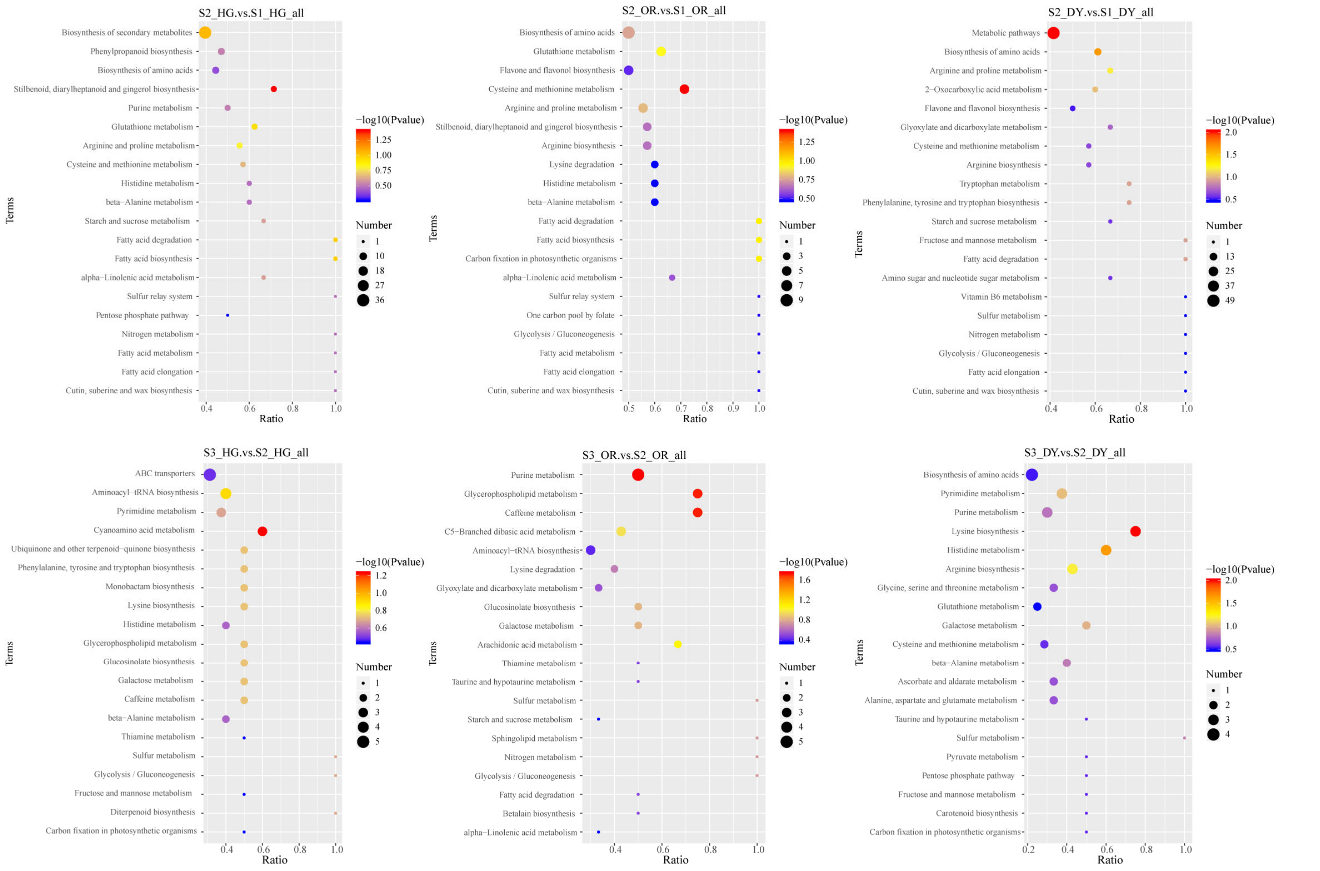

Figure S2. KEGG pathway enrichment analysis of differential metabolites in comparison groups S2vsS1 and S3vsS2. The horizontal coordinate of the graph is the ratio of the number of differential metabolites annotated to the KEGG pathway to the total number of differential metabolites, and the vertical coordinate is the KEGG pathway. The size of the dots represents the number of metabolites annotated to the KEGG pathway, and the color gradient from red to purple represents the significance of the size of enrichment. Sample 1 vs Sample 2 represents the different metabolites in the former compared to the latter.

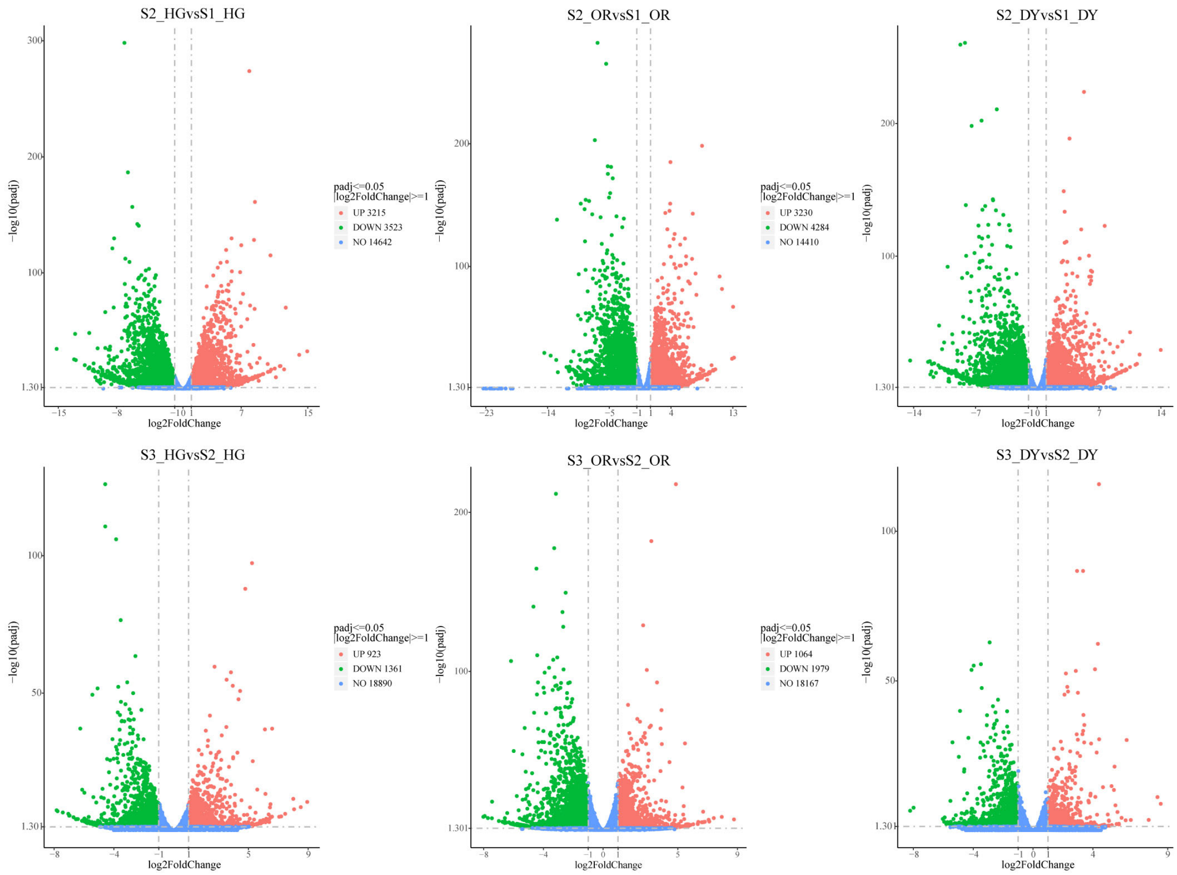

Figure S3. Volcano plots of DEGs across different groups. The red dots, inns, and blue dots represent up-regulated, down-regulated, and non-significant DEGs, respectively. The horizontal axis represents fold change in gene expression levels, and the vertical axis represents the significance of differences.

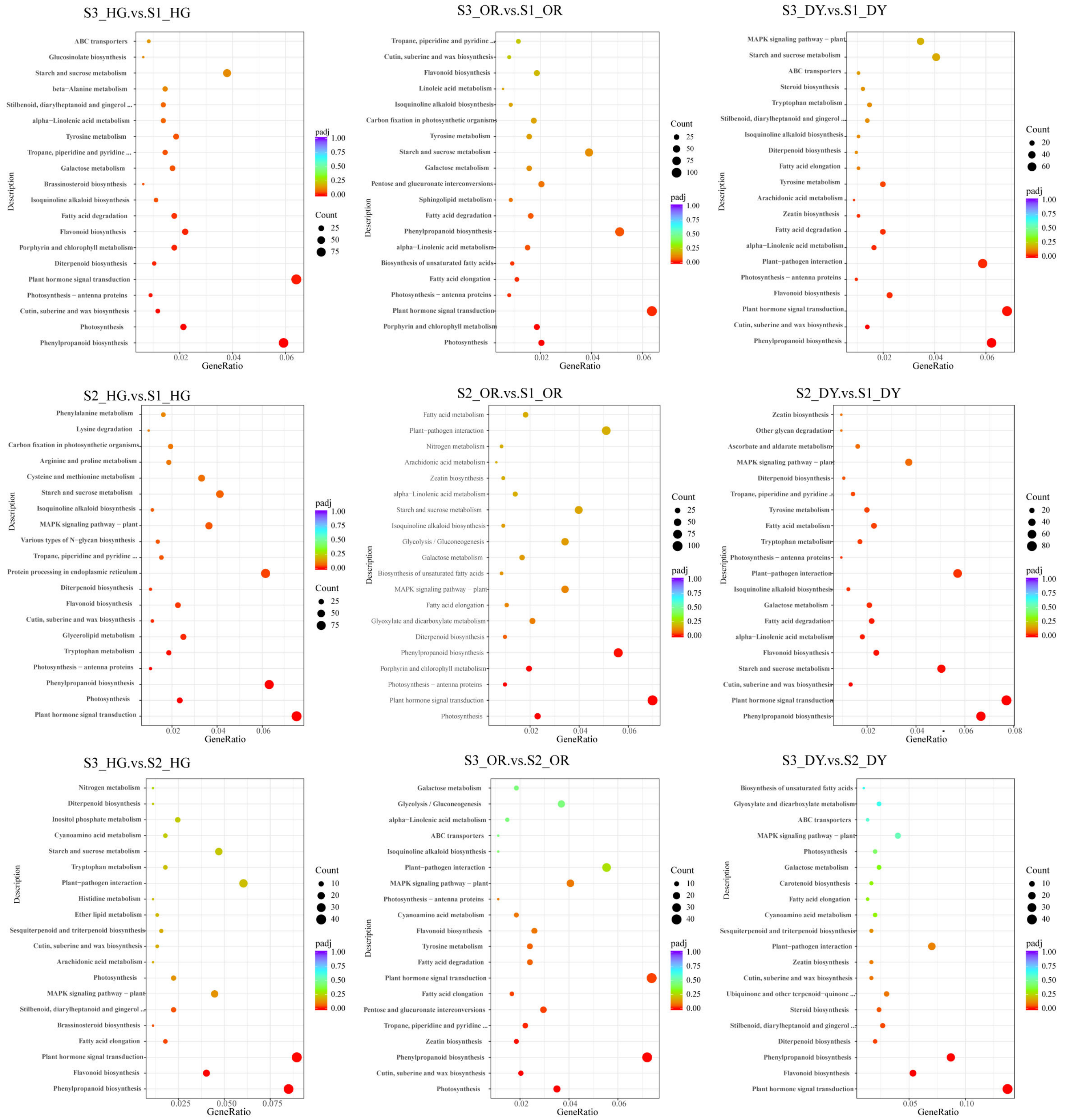

Figure S4. Results of KEGG enrichment for differentially expressed genes in nine comparison groups. The horizontal coordinate of the graph is the ratio of the number of differential genes annotated to the KEGG pathway to the total number of differential genes, the vertical coordinate is the KEGG pathway, the size of the dot represents the number of genes annotated to the KEGG pathway, and the color from red to purple represents the significance size of enrichment.

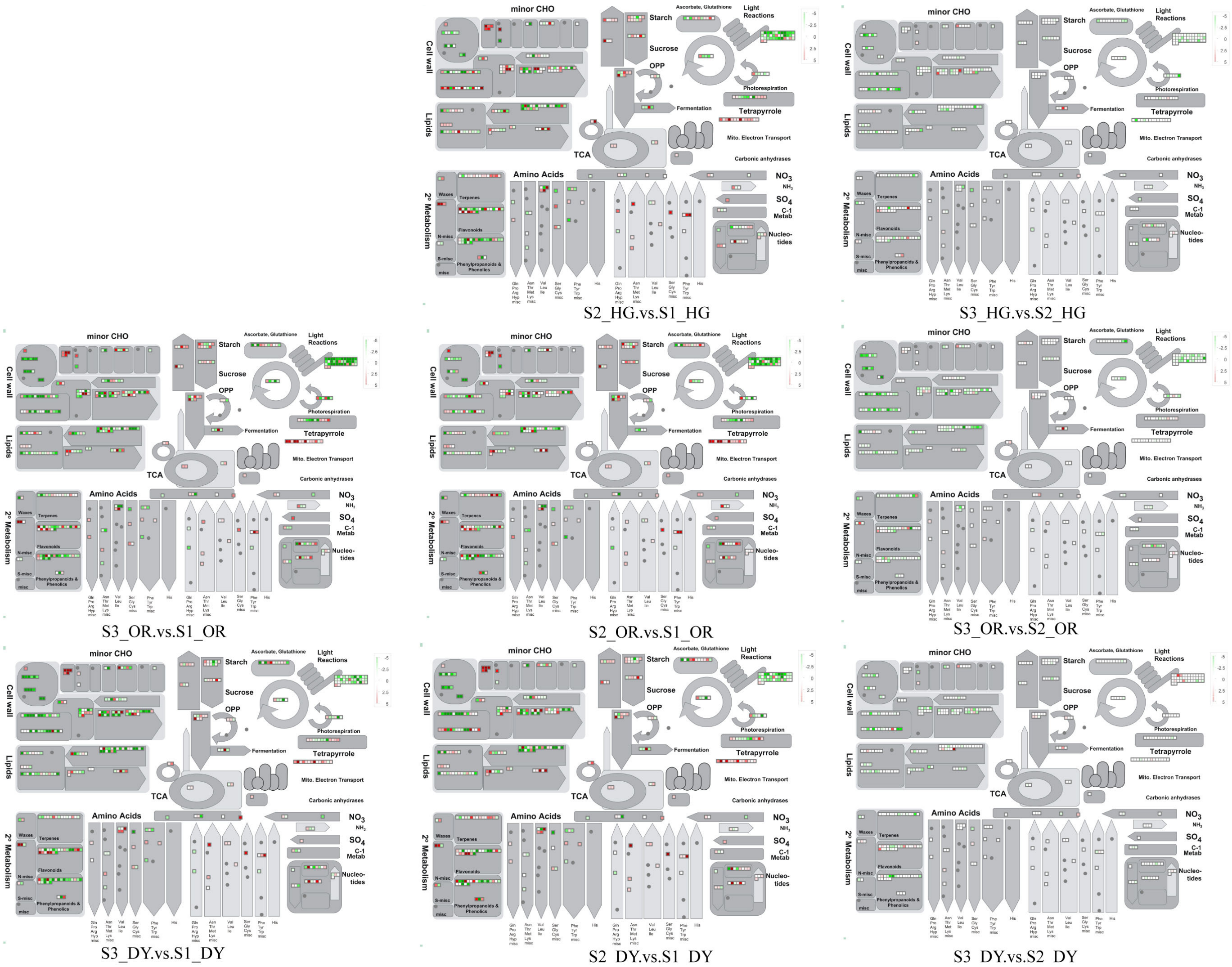

Figure S5. MapMan-based "Metabolic Overview" diagram for the remaining 8 groups of DEGs except for the "S3_HG.vs.S1_HG" group. See Figure 7a in this paper for an explanation of the colors in this graph.

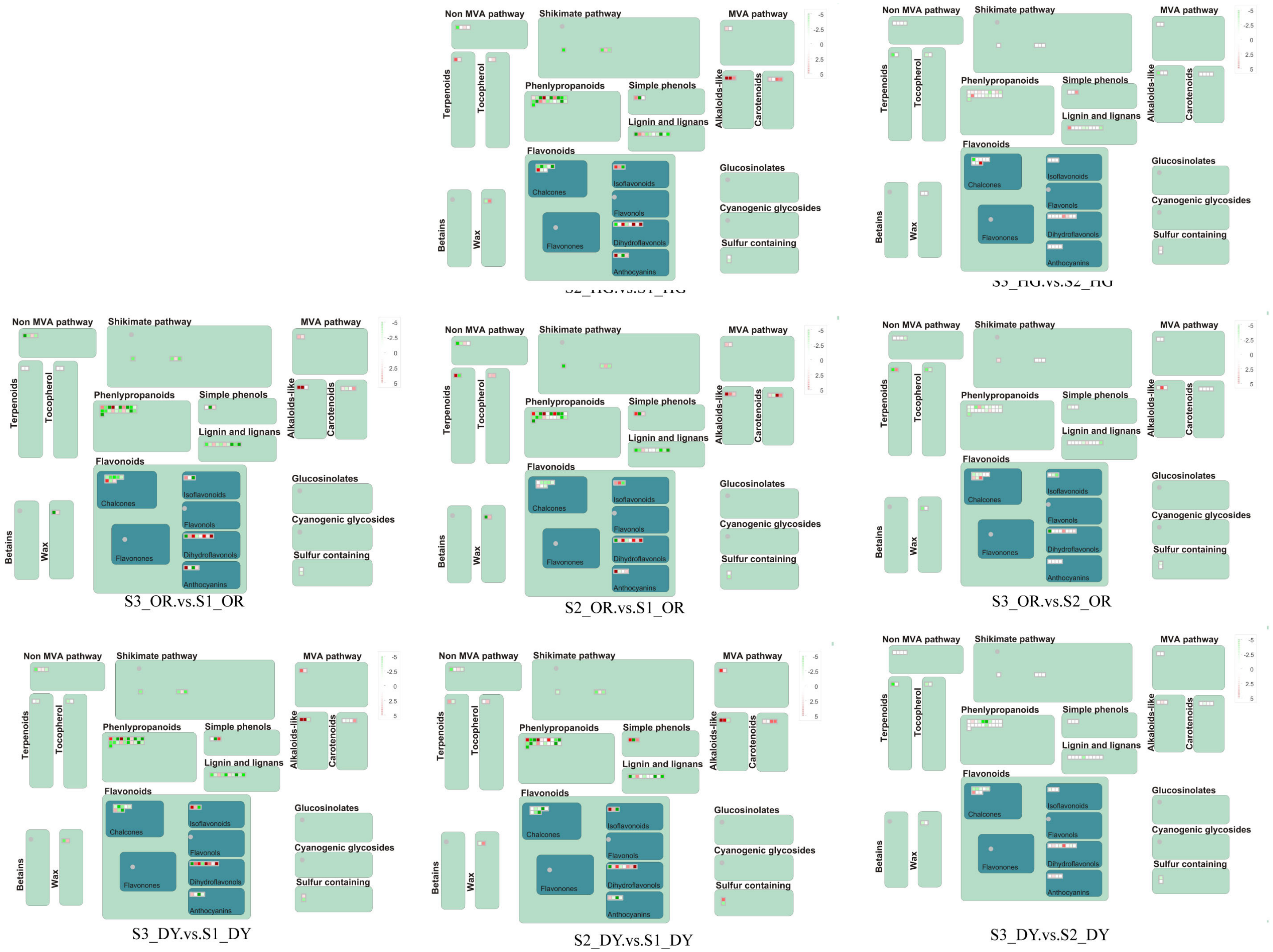

Figure S6. MapMan-based overview map of "Secondary metabolism" for the remaining 8 groups of DEGs except for the "S3_HG.vs.S1_HG" group. See Figure 8a in this paper for an explanation of the colors in this graph.

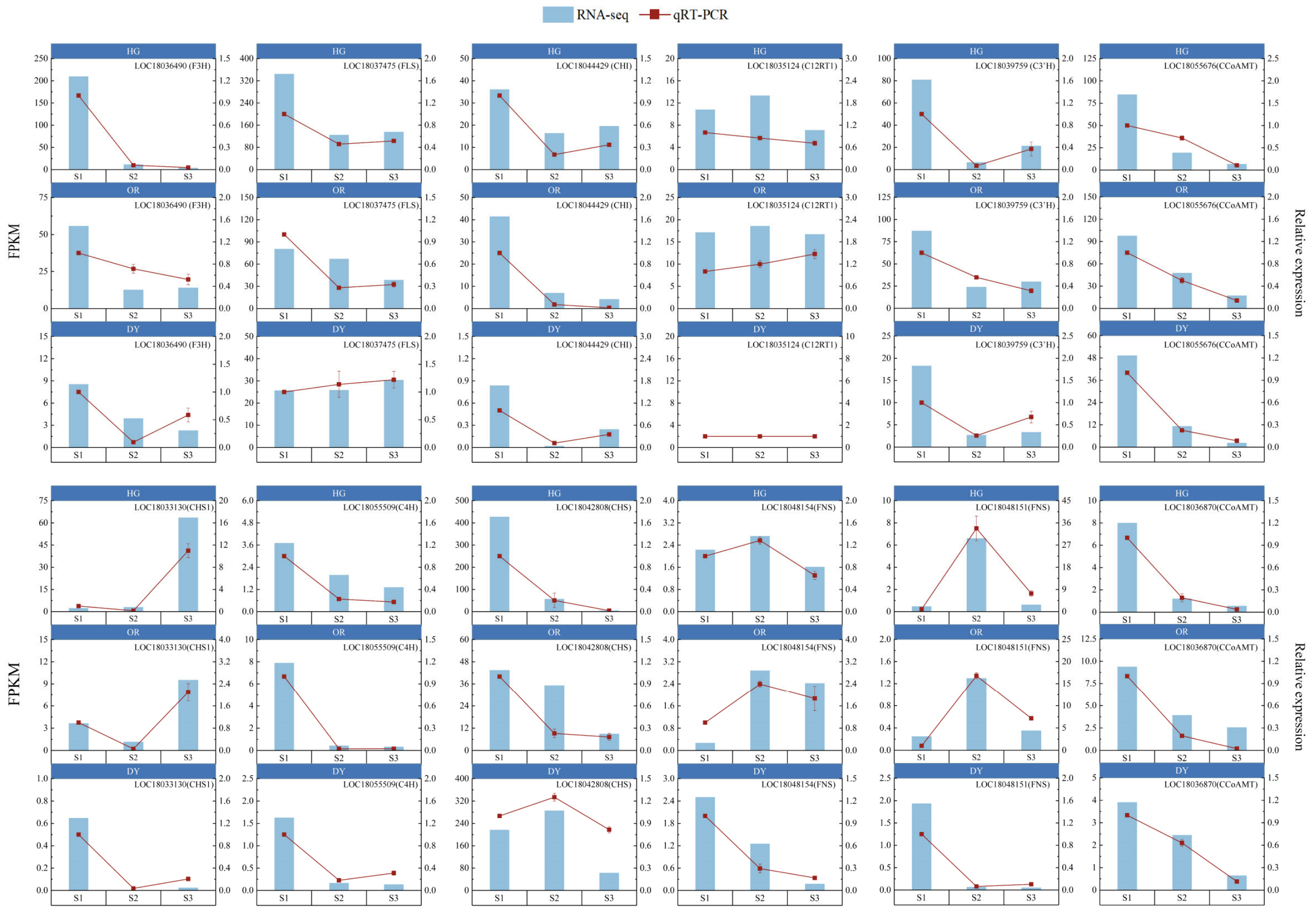

Figure S7. Quantitative RT-PCR analysis was performed on selected DEGS based on the results of RNA sequencing analysis. The left y-axis shows the expression data from RNA-seq (blue histogram) analysis. The right y-axis indicates the relative gene expression levels of the corresponding qRT-PCR analysis (red line). x-axis indicates samples from different growth and development periods.

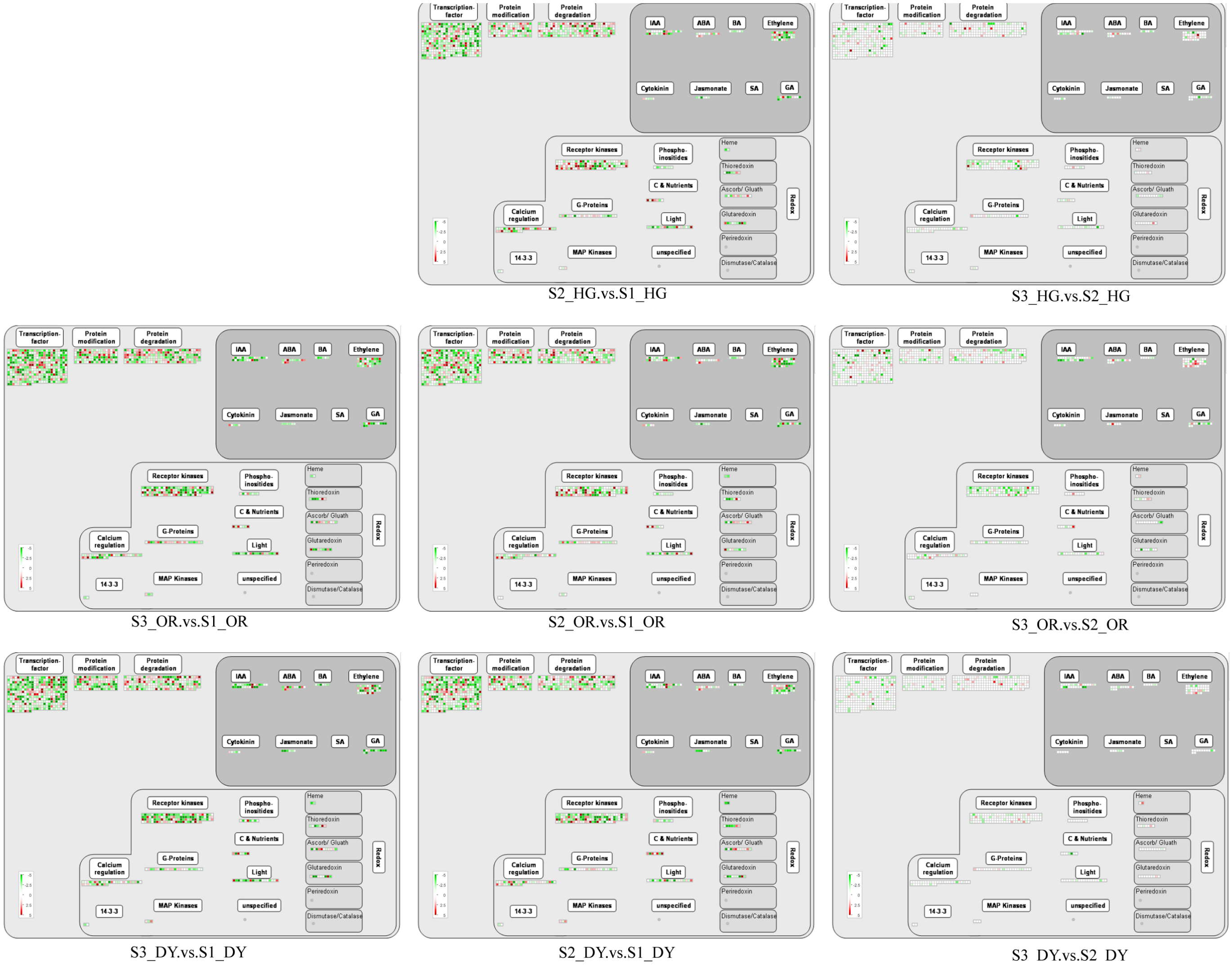

Figure S8. MapMan-based "regulatory overview" plot of the remaining 8 groups of DEGs, except for the "S3_HG.vs.S1_HG" group. The interpretation of the colors in this figure is shown in Figure 9a of this paper.

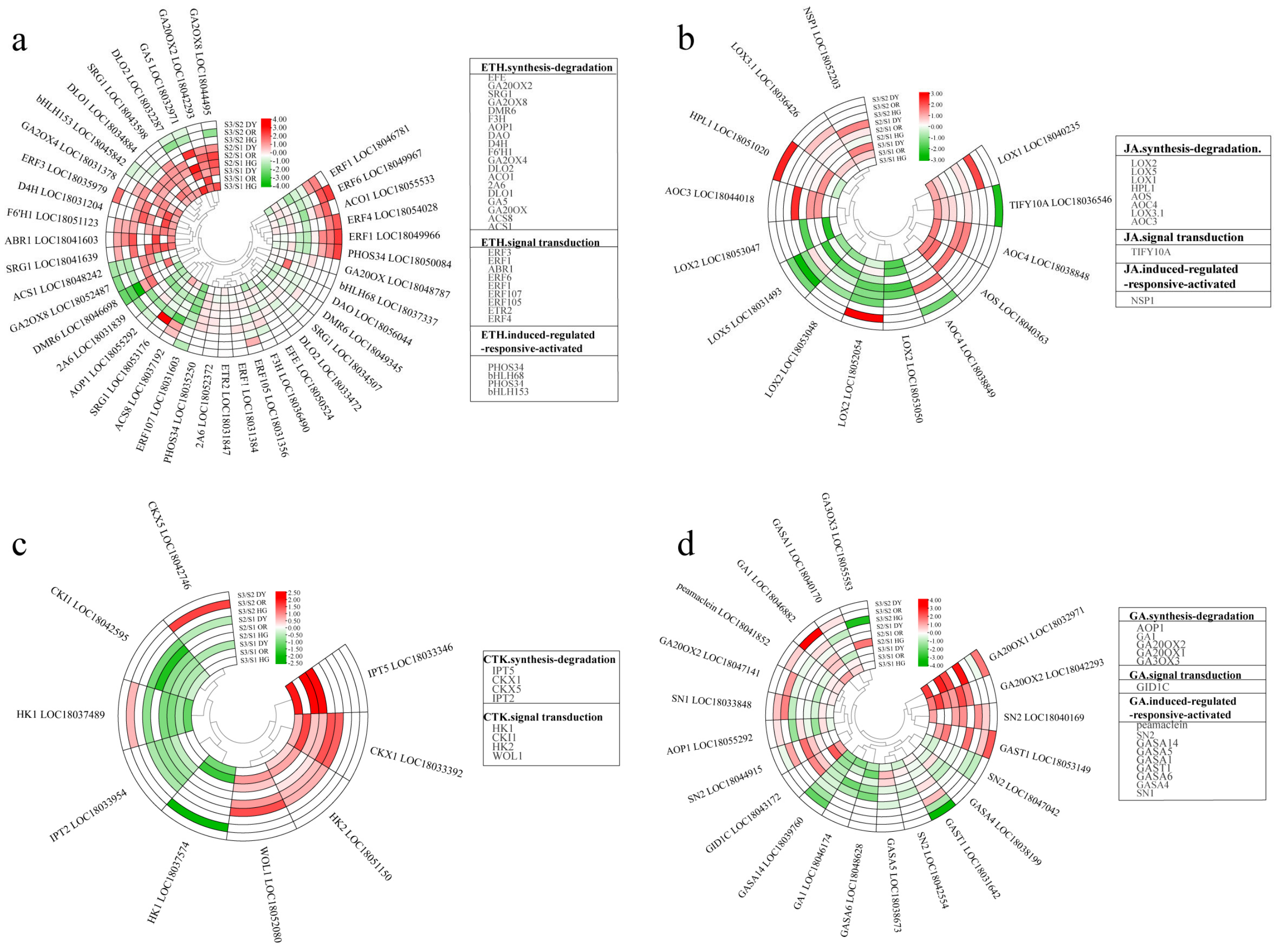

Figure S9. Plant hormone-related differentially expressed genes during citrus development and ripening: (a) ethylene; (b) jasmonic acid; (c) cytokinin; (d) gibberellin. Red and green represent up- and down-regulation, respectively.

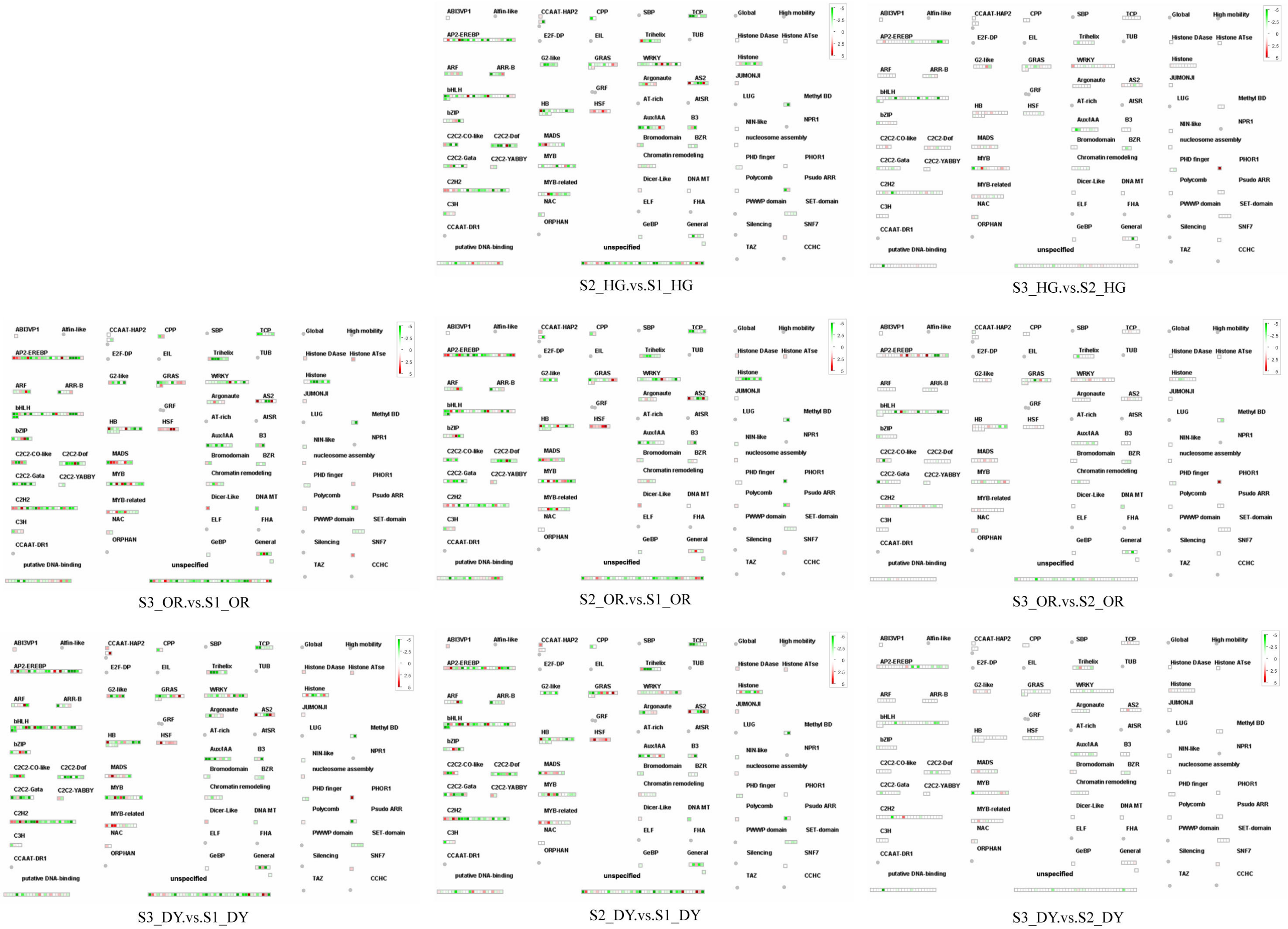

Figure S10. MapMan-based " Regulation" module diagram for the remaining 8 groups of DEGs, except for the "S3_HG.vs.S1_HG" group. The interpretation of the colors in this figure is shown in Figure 10a of this paper.
